# The journey to diagnosis of wild-type transthyretin-mediated (ATTRwt) amyloidosis: a path with multisystem involvement

**DOI:** 10.1186/s13023-024-03407-3

**Published:** 2024-11-08

**Authors:** Chafic Karam, Colleen Moffit, Catherine Summers, Madeline P. Merkel, Fran M. Kochman, Laure Weijers, Mathilde Puls, Marieke Schurer, Emily Jones, Nicola Mason, Muriel Finkel, Paula Schmitt, Mazen Hanna

**Affiliations:** 1 Department of Neurology, University of Pennsylvania, Philadelphia, USA; 2 Alnylam Pharmaceuticals, Cambridge, MA USA; 3 Lumanity, Utrecht, The Netherlands; 4 Lumanity, London, UK; 5 Lumanity, Manchester, UK; 6 Amyloidosis Support Groups Inc., Wood Dale, IL USA; 7 Department of Cardiovascular Medicine, Cleveland Clinic, Cleveland, OH USA

**Keywords:** Hereditary transthyretin-mediated amyloidosis, Wild-type transthyretin-mediated amyloidosis, hATTR, wtATTR, ATTRwt, ATTRv, Amyloidosis, Diagnosis, Patient journey

## Abstract

**Background:**

Wild-type and hereditary transthyretin-mediated amyloidosis (ATTRwt and ATTRv amyloidosis, respectively) are progressive, fatal diseases with a broad range of clinical presentations and multisystem effects. Despite having a higher prevalence, ATTRwt amyloidosis is less well characterized due to its non-hereditary nature, and its relatively poorer disease awareness delays diagnosis. Understanding of its natural history has evolved in recent years, but this is largely based on physician-collected data rather than patients’ reports of their own experiences. A mixed methods approach was used to evaluate how the healthcare journeys of patients with ATTRv and ATTRwt amyloidosis compare.

**Methods:**

A quantitative survey was administered to US-patients diagnosed with both ATTRwt amyloidosis and ATTRv amyloidosis identified through a patient support group. Subsequent in-depth interviews with participants with ATTRwt amyloidosis were conducted. Quantitative data with related qualitative quotes from patients were produced to characterize their paths to diagnosis and the disease burden experienced.

**Results:**

A total of 47 respondents completed the survey (ATTRv, n = 20 and ATTRwt, n = 27) and a total of 14 survey respondents with ATTRwt amyloidosis were interviewed. Survey results reported a high disease burden for patients with both conditions, with patients with ATTRwt amyloidosis reporting more diagnoses and procedures prior to their final diagnosis. Interviews with participants with ATTRwt amyloidosis revealed that patients face a high symptomatic burden of disease. Diagnosis was often delayed due to three key factors: (1) early signs of ATTRwt amyloidosis were often assumed to be related to old age; (2) many medical specialists working in silos were involved in participants’ diagnostic; and (3) there was a general lack of disease awareness. Early indicators such as carpal tunnel syndrome were often overlooked. Participants were typically diagnosed after the disease had progressed to include severe cardiac symptoms such as atrial fibrillation and severe shortness of breath. Sleep apnoea was also reported by a number of participants, with a considerable impact on quality of life.

**Conclusions:**

Our study provides insight into the overall impact of the patient journey on their quality of life and demonstrates how increased awareness of ATTRwt amyloidosis and more coordinated engagement with physicians could reduce the time to diagnosis.

**Supplementary Information:**

The online version contains supplementary material available at 10.1186/s13023-024-03407-3.

## Introduction

Wild-type transthyretin-mediated amyloidosis (ATTRwt amyloidosis) is a non-hereditary, progressive, debilitating, fatal disease caused by the accumulation of misfolded wild-type transthyretin (TTR) amyloid fibrils. Hereditary ATTR amyloidosis (ATTRv; v for variant) is caused by inherited *TTR* gene variants, leading to unstable TTR that subsequently misfolds and accumulates in multiple organs and tissues. The natural history of ATTRwt amyloidosis has only recently been catalogued, while ATTRv amyloidosis has been better characterized [1, 2]. The worldwide prevalence of ATTRwt amyloidosis is estimated to be around 200,000–300,000 [3, 4], while ATTRv amyloidosis affects approximately 50,000 people across the globe [5, 6]. Median survival for ATTRv amyloidosis depends on the variant and ranges from 2.5 to 10 years; in contrast, the survival of people with ATTRwt amyloidosis ranges between 2.5 and 5.5 years [7–16].

ATTR amyloidosis, either hereditary or wild-type, is a multisystem disease with a wide variety of clinical presentations, often involving the heart, sensorimotor system, autonomic nervous system and musculoskeletal tissues [17]. For ATTRwt amyloidosis, symptoms typically present after 60 years of age, with an average age at diagnosis of 75 years. It is typically diagnosed when patients develop cardiomyopathy [12]. Until recently, a diagnosis of ATTRwt amyloidosis was often made once the disease has progressed to heart failure, but recent data suggest that musculoskeletal-related symptoms such as carpal tunnel syndrome, lumbar spinal stenosis and biceps tendon rupture can occur years before cardiac involvement [18–21]. The wide variety of symptoms and the progressive, debilitating nature of ATTRwt amyloidosis results in a rapid decline in physical function and quality of life [22, 23].

Early detection and treatment reduce symptom burden and disease progression [22, 24, 25]. However, early detection is rare, especially in ATTRwt amyloidosis given its non-hereditary nature. Awareness of amyloidosis among healthcare practitioners (HCPs) remains low, despite recent improvements. Part of the challenge to earlier diagnosis is that systemic ATTRwt amyloidosis symptoms are heterogeneous and non-specific, meaning they are often attributed to other chronic or age-related diseases. Furthermore, disease manifestations may be widespread and treated in silos by multiple specialists, who may not recognize the totality of the multisystem clues that are characteristic of this condition. In the US, ATTRwt amyloidosis was commonly diagnosed at only a handful of expert centres; as a result, diagnosis is frequently missed or delayed for several years [26, 27].

While the understanding of the natural history of ATTRwt amyloidosis has evolved, most research has been based on physician-collected data sources. Examples of such sources include retrospective chart reviews, electronic health record analyses, and surveys of comorbidities [12, 28]. To date, little research has been conducted with patients directly describing: (1) their experiences with the early signs and symptoms of ATTRwt amyloidosis; (2) their timing in relation to patients’ formal diagnosis; and (3) the impact of these earlier manifestations on patients’ lives [1]. This is in contrast with ATTRv amyloidosis, where genetic testing and the familial nature of the condition have facilitated better detection and understanding of disease progression [29].

The objective of our study was to understand the first-hand patient experience of ATTRwt amyloidosis, how it differs from that of ATTRv amyloidosis, and the key factors that lead from symptom onset to eventual diagnosis. We also gathered data on the overall disease burden and symptomatology of ATTRwt amyloidosis, with the aim of raising awareness of the disease among HCPs and help them more readily recognize the early signs and symptoms of ATTRwt amyloidosis to quicken the diagnostic process.

## Methods

A mixed-methods approach was used for this study, incorporating quantitative and qualitative data gathered from a survey of people in the US with ATTR amyloidosis and subsequent interviews. Participants were members of Amyloidosis Support Groups (ASG), a US-based non-profit organization that maintains and facilitates support group meetings for those impacted by this disease [30]. Participants completed a cross-sectional, web-based survey and then continued on to in-depth interviews. All study materials—including the study protocol, consent forms, and the survey and interview guides—were developed and approved by all members of the study team and ASG. The study protocol and interview guide are available in Additional files 1 and 2, respectively. The Western Copernicus Group Institutional Review Board approved both phases of the study (reference number: 1297323).

### Participants and data collection

Between 30 November and 8 December 2020, members of the ASG who self-identified as having ATTRwt or ATTRv amyloidosis were sent an online survey to gather information on their experiences of the disease. Questions were included about participants’ earliest symptoms commonly associated with ATTR amyloidosis up to receiving a formal diagnosis. Survey participants with ATTRwt amyloidosis were then invited for in-depth interviews. The interviews focused on patients with ATTRwt amyloidosis because of the lack of knowledge within the current literature.

The survey collected data on participant demographics, medical history and the patient experience during the period prior to ATTR amyloidosis diagnosis. Medical history included signs, symptoms and diagnoses. Signs refer to what an HCP finds or measures when examining a patient, while symptoms refer to what a patient reports to an HCP. Diagnoses are defined as procedures related to orthopaedic, cardiac or other manifestations associated with ATTR amyloidosis and related HCP interactions. After completing the survey, participants could express interest in participating in a further telephone/video-conference interview if they met the interview inclusion criteria, which were as follows:

- People aged 18 and over with a confirmed diagnosis of ATTRwt amyloidosis from a medical professional who:
  - Were able to provide written informed consent
  - Were able to participate in telephone interviews
  - Had the cognitive and linguistic skills necessary to participate
  - Completed the survey and included information on type of manifestation, age at which manifestation occurred, and type of HCP seen for each manifestation

Following the survey, semi-structured 1-h interviews were conducted to further understand each participant’s journey to a diagnosis of ATTRwt amyloidosis. Information shared by the participants was recorded, transcribed and anonymized.

Interview topics included symptoms, diagnoses, and HCP visits or procedures that participants had experienced before receiving their formal diagnosis of ATTRwt amyloidosis. Retrospective questions were included to understand how the participants’ journey impacted different domains such as their ability to work, their social life, and their physical and emotional wellbeing. Further questions were posed to understand which HCPs were involved during their journey to diagnosis, including how their interactions helped address unmet needs and assisted in reaching their diagnosis of ATTRwt amyloidosis. The interview guide is available in Additional file 2.

### Data analysis

**Fig. 1:**
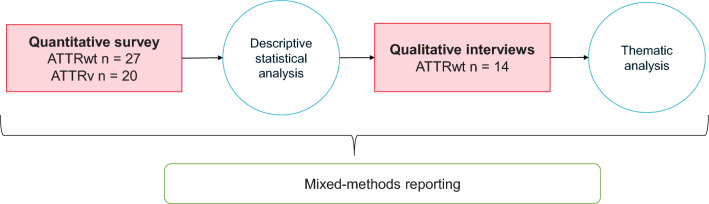
Schematic overview of study components. ATTRv, Hereditary transthyretin; ATTRwt, Wild-type transthyretin

A visual overview of the study design is provided in Fig. 1.

Closed-ended survey questions were analysed using descriptive statistics. Means, medians, ranges, and standard deviation values were calculated. When only ranges were provided, mid-points were used. Where question-specific data were missing due to non-response, the denominator was adjusted to include only respondents.

In the survey, participants reported what different manifestations they considered to be related to their ATTR amyloidosis, as well as their various interactions with HCPs before receiving a formal diagnosis. To detect patterns between participants, reported signs and symptoms as well as procedures, diagnoses, and HCP interactions were sorted into three time-based categories: 0–3, 4–10, and over 10 years pre-ATTR amyloidosis diagnosis. ATTRwt amyloidosis manifestations were subsequently grouped by clinical experts into three categories: orthopaedic, cardiovascular, and other (not cardiovascular or orthopaedic). These groups of manifestations were used as an analysis scheme to present results, but should not be taken to represent clinically valid aetiologies of each manifestation. A separate category was initially considered for neuropathy-related manifestations, to capture symptoms that were neither orthopaedic nor cardiovascular related. However, we considered that the aetiology of certain manifestations such as fatigue and sleep apnoea could not be unambiguously categorized as neuropathy related. To avoid complicating the stratification of results for the analysis, a decision was made to retain the three aforementioned categories and to group fatigue, sleep apnoea and neuropathy-related symptoms under ‘others’ (i.e., not cardiovascular or orthopaedic related). Finally, the most prevalent manifestations were investigated, with the term ‘prevalent’ being applied when 10% or more of unique respondents reported a particular manifestation.

In preparation for the in-depth interviews, individual journey maps were prepared to guide participants (a template of patients’ unique diagnostic journey map is available in Additional file 3). Each map provided a chronological overview of when a participant reported certain manifestations and with which HCP(s) they interacted.

Two independent members of the research team developed a coding framework in Microsoft Excel® to reduce researcher bias. The interview data were then thematically analysed using NVivo®, applying the coding framework to all interview transcripts. For the analysis, deductive and inductive coding were used, and any emerging themes were discussed between the researchers.

Personal information and raw study materials were handled in accordance with the EU General Data Protection Regulation.

## Results

### Participant characteristics

**Fig. 2:**
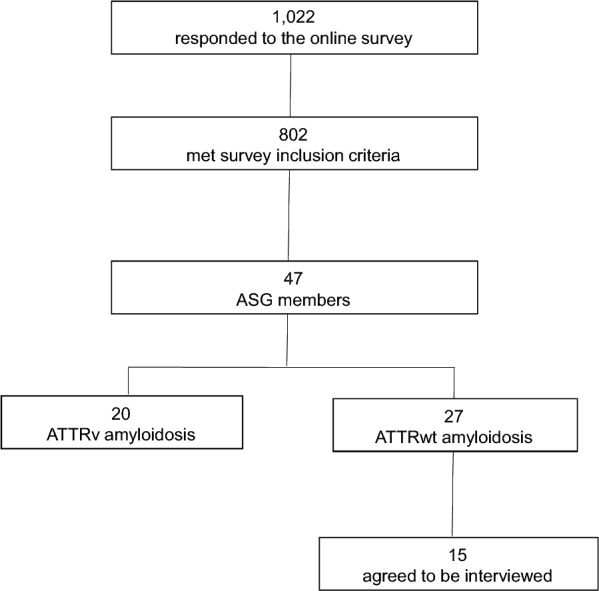
Flow chart illustrating the recruitment process. ASG, Amyloidosis Support Groups; ATTRv, Hereditary transthyretin; ATTRwt, Wild-type transthyretin

There were 1022 responses to the online survey, 802 of which met the survey inclusion criteria. After further screening, 47 respondents were determined to be ASG members: 20 had ATTRv amyloidosis, and 27 had ATTRwt amyloidosis. People with ATTRwt amyloidosis were invited for interviews, and a total of 14 people with ATTRwt amyloidosis agreed to be interviewed (Fig. 2). Participant characteristics for survey respondents and interview participants are presented in Tables 1 and 2, respectively.

**Table 1:** Survey respondent characteristics

Participant characteristics	ATTRv amyloidosis n = 20 (%)	ATTRwt amyloidosis n = 27 (%)
Gender	M: 9 (45)	M: 24 (89)
	F: 11 (55)	F: 3 (11)
Current age, mean	65.6 years	72.5 years
Age groups	≤ 40: 0 (0)	≤ 40: 0 (0)^a^
	41–50: 0 (0)	41–50: 1 (4)
	51–60: 6 (30)	51–59: 0 (0)
	61–70: 10 (50)	60–70: 8 (30)
	> 71: 4 (20)	≥ 71: 18 (67)
Age at diagnosis, mean	60.1 years	69.9 years
Median	60 years	70 years
Range	42–79	46–82
Years since diagnosis, mean	Mean years: 5.5 years	Mean years: 2.6 years
Median	Median years: 2.5	Median years: 2
Range	Range: 0–26	Range: 0–11
Ethnic background		
White	17 (85)	23 (85)
Asian	1 (5)	1 (4)
Black or African American	2 (10)	2 (7)
Other	0	1 (4)^b^
Diagnosis of polyneuropathy and/or cardiomyopathy		
Cardiomyopathy	3 (15)	17 (63)
Polyneuropathy	9 (45)	1 (4)
Both polyneuropathy and cardiomyopathy	7 (35)	7 (26)
I don’t know/neither/blank	1 (5)	2 (7)

ATTRv amyloidosis, Hereditary transthyretin-mediated amyloidosis; ATTRwt amyloidosis, Wild-type transthyretin-mediated amyloidosis

^a^Please note that for ATTRwt amyloidosis, the sum of age groups equals 101% due to rounding with the following percentages: ASG members: 3.7%, 29.6%, and 66.7%

^b^One participant responded ‘American’ to this question

**Table 2:** Interview participant characteristics

Demographics and characteristics	Participants with ATTRwt amyloidosis (N = 14) (%)
Gender	
Male	11 (76)
Female	3 (21)
Age at interview, mean [range]	74 [62–85]
Age at diagnosis, mean [range]	69.5 [59–82]
Years since diagnosis, mean [range]	4.6 [2–13]
Negative *TTR* genetic test	
Yes^a^	14 (100)
Age at genetic test, years, mean [range]	69.7 [59–82]
Diagnosing physician	
Cardiologist	12 (86)
Haematologist	1 (7)
Other^b^	1 (7)
Ethnicity	
White	13 (93)
Asian	1 (7)
Work status	
Retired	13 (93)
Long-term disability	1 (7)
Marital status	
Married	11 (79)
Partnership	3 (21)

ATTRwt amyloidosis, Wild-type transthyretin-mediated amyloidosis

^a^One participant was diagnosed with ATTRwt amyloidosis through a heart biopsy and never had the genetic test

^b^On the recommendation of the amyloidosis specialist, one participant indicated being diagnosed at the Mayo Clinic through a heart biopsy, which determined the diagnosis of ATTRwt amyloidosis

### Survey results

#### Journey to diagnosis

According to survey results, the mean age of diagnosis was 60.1 years (median: 60 years; range: 42–79 years) for respondents with ATTRv amyloidosis and 69.5 years (median: 70 years; range: 46–82) for survey respondents with ATTRwt amyloidosis. To estimate the journey length of patients and consider the potential delay to diagnosis, we used the time between: (1) the age at which participants reported to have experienced their first symptom suspected to be related to amyloidosis or procedure relating to this potential symptom; and (2) the year of their formal ATTR amyloidosis diagnosis. For the former value, participants were presented with a list of symptoms and procedures to select from and report the age at which they had experienced the symptom or undergone the procedure (see Additional file 4 for the predefined list of symptoms and procedures). Based on this approach, participants with ATTRv amyloidosis were diagnosed with a potential median delay of 13 years (range: 0–35). Participants with ATTRwt amyloidosis had a potential median delay of 16 years (range: 2–32 years; one participant's result was omitted as an outlier, as it was reported to be 58 years due to a shoulder dislocation at 20 years old).

**Fig. 3:**
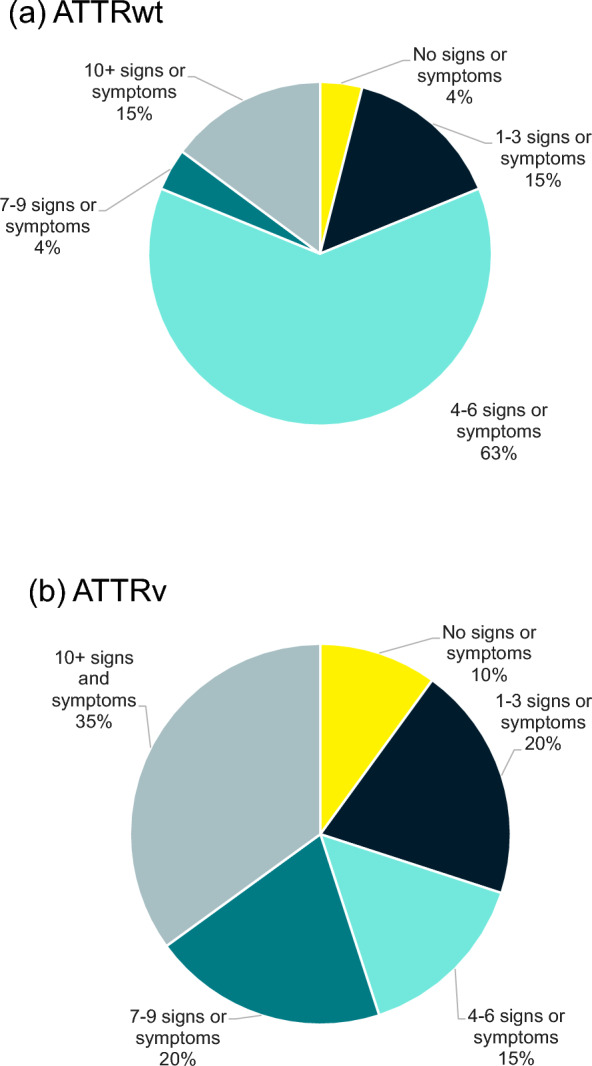
Percentage of survey respondents who experienced 0–10 + signs or symptoms before diagnosis. ATTRv, hereditary transthyretin; ATTRwt, wild-type transthyretin. *Notes*: Percentage of survey respondents who experienced 0–10 + signs or symptoms before diagnosis with **a** ATTRwt amyloidosis (n = 27) and **b** ATTRv amyloidosis (n = 20)

The initial number of signs and symptoms differed between respondents with ATTRv and ATTRwt amyloidosis. However, both conditions demonstrated progressive disease burden over time. Fifteen percent and 63% of respondents with ATTRv and ATTRwt amyloidosis, respectively, reported experiencing at least 4–6 symptoms in the period before ATTR amyloidosis diagnosis. However, people with ATTRv amyloidosis experienced a greater number of symptoms, with 35% of people with ATTRv amyloidosis reporting more than 10 signs or symptoms. Figure 3a, b present the number of signs and symptoms experienced by amyloidosis type.

**Fig. 4:**
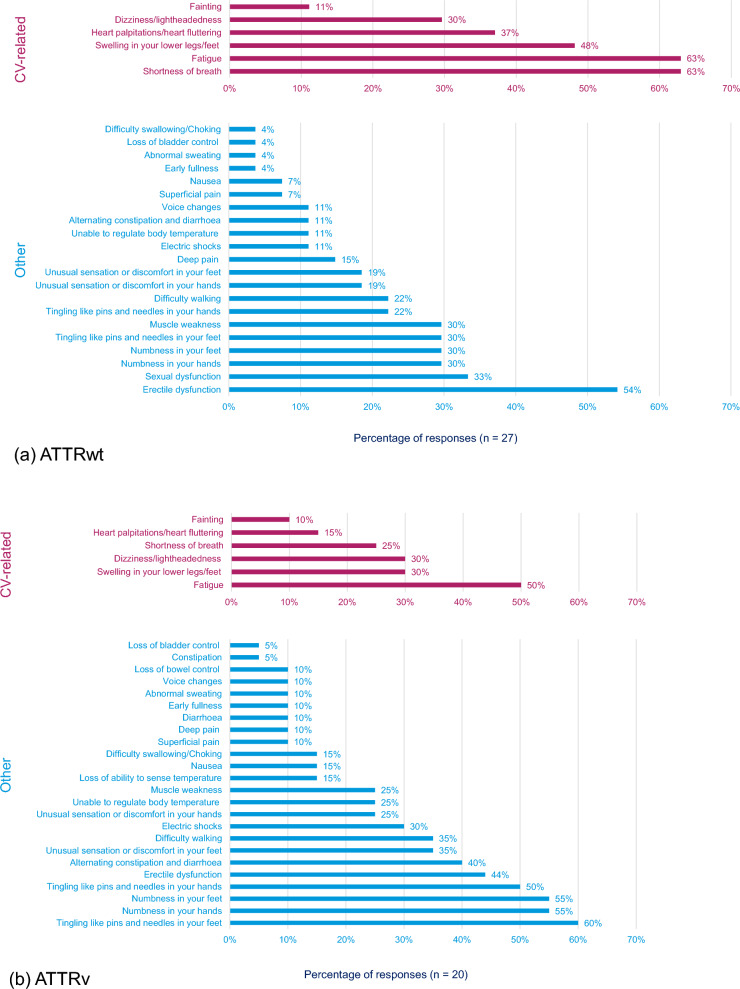
Prevalence of signs or symptoms before diagnosis. ATTRv, Hereditary transthyretin; ATTRwt, Wild-type transthyretin. *Notes*: Prevalence of signs or symptoms before diagnosis with **a** ATTRwt amyloidosis (n = 27) and **b** ATTRv amyloidosis (n = 20). When analysing the prevalence of ‘Erectile dysfunction’ and ‘Sexual dysfunction’, we have used the denominator of male respondents (ATTRwt, n = 24; ATTRv, n = 9) and female respondents (ATTRwt, n = 3; ATTRv, n = 0), respectively

Prior to diagnosis, the most prevalent respondent-reported symptom in people with ATTRwt amyloidosis was shortness of breath (63%) (Fig. 4a). Numbness (55%) and/or tingling-like pins and needles in hands (50%) and feet (60%) were the most common signs/symptoms reported in people with ATTRv amyloidosis (Fig. 4b). Fatigue was frequently reported as a symptom associated with their condition by both groups of respondents. When considering signs or symptoms more than 10 years before diagnosis, erectile dysfunction was the only symptom reported in 10% or more of male respondents with ATTRwt amyloidosis.

**Fig. 5:**
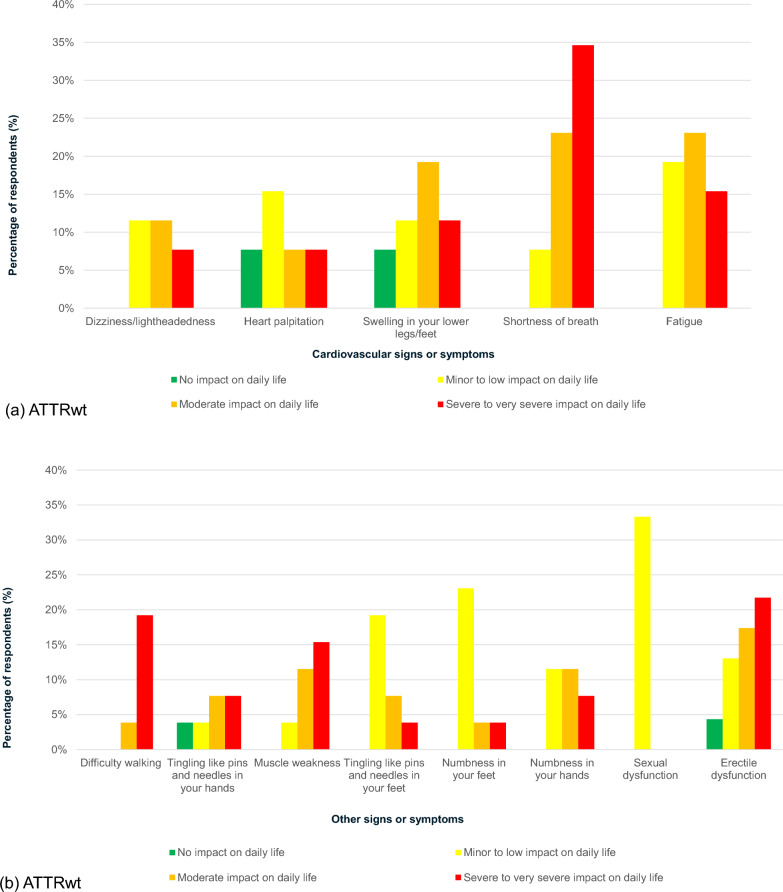

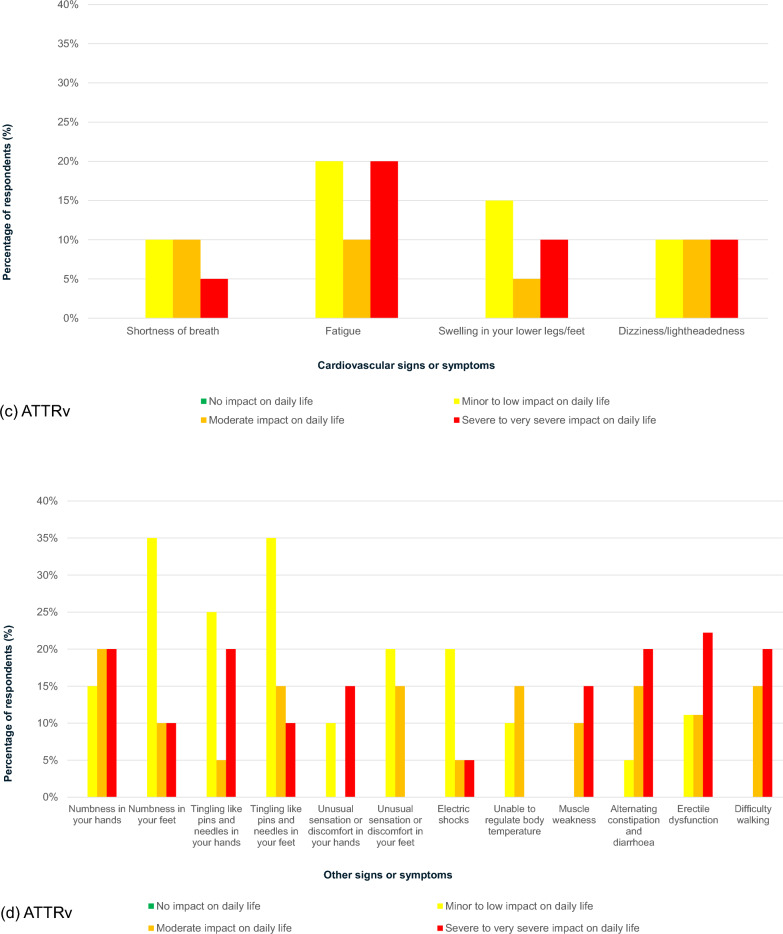
Impact of prevalent signs or symptoms (≥ 20%) in survey participants. ATTRv, Hereditary transthyretin; ATTRwt, Wild-type transthyretin. *Notes*: Impact of prevalent signs or symptoms (≥ 20%) in survey participants: **a** ATTRwt (n = 26), cardiovascular-related; **b** ATTRwt (n = 26), other, non-cardiovascular-related signs and symptoms; **c** ATTRv (n = 20), cardiovascular-related; **d** ATTRv (n = 20), other, non-cardiovascular-related signs and symptoms. The graph reports impact of cardiovascular-related and other, non-cardiovascular-related signs and symptoms that were prevalent in 20% or more of the population. One male respondent in the ATTRwt group skipped the question. For the other symptoms, when analysing the prevalence of erectile dysfunction/sexual dysfunction, the denominator used was out of either male respondents only (for erectile dysfunction; n = 23 for ATTRwt and 9 for ATTRv) or female respondents only (for sexual dysfunction; n = 3 for ATTRwt and 0 for ATTRv)

People with both ATTRv and ATTRwt amyloidosis experienced severe to very severe impact on their daily lives due to their symptoms (Fig. 5a, d), most of which occurred between 0 and 4 years before their diagnosis. Shortness of breath was the most burdensome of the most prevalent cardiovascular symptoms in people with ATTRwt amyloidosis, followed by fatigue. Erectile dysfunction (among men), difficulty walking and muscle weakness were the most burdensome non-cardiovascular-related signs or symptoms. Respectively, 22%, 19% and 15% of respondents with these symptoms experienced a severe to very severe impact on their daily life.

Burdensome cardiovascular-related signs or symptoms present in 20% or more of ATTRv amyloidosis respondents were fatigue (20%), followed by swelling in lower legs/feet (10% and dizziness/light-headedness (10%). For each of these, respondents reported being severely to very severely impacted by these symptoms. The most prevalent symptoms categorized as non-cardiovascular for the purpose of this study were as follows: erectile dysfunction (22%) (among men) was the most burdensome, followed by numbness in hands (20%), alternating constipation and diarrhoea (20%), difficulty walking (20%), and tingling pins and needles in the hands (20%). Respondents reported being severely to very severely impacted by these signs and symptoms.

#### Other diagnoses

**Fig. 6:**
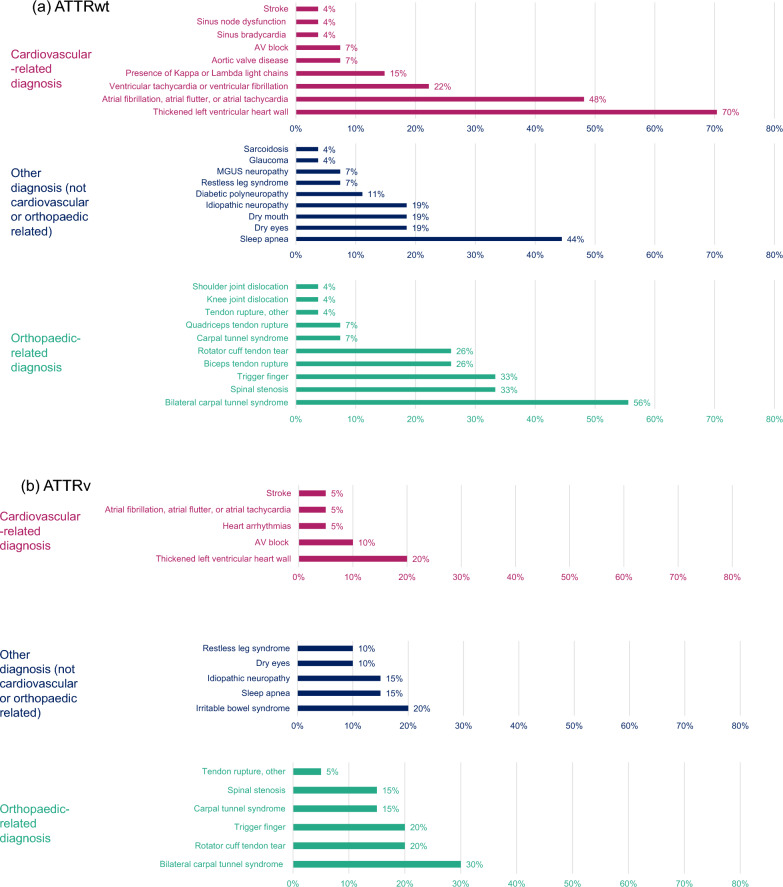
Prevalence of other diagnoses received by survey respondents. ATTRv, Hereditary transthyretin; ATTRwt, Wild-type transthyretin; AV, Atrioventricular; MGUS, Monoclonal gammopathy of undetermined significance. *Notes*: Prevalence of other diagnoses received by survey respondents with **a** ATTRwt (n = 27) and **b** ATTRv (n = 20)

Many survey respondents reported being diagnosed with other diseases prior to their ATTR amyloidosis diagnosis. Those with ATTRwt amyloidosis experienced a greater number of other diagnoses than those with ATTRv amyloidosis (Fig. 6a, b). The most common other diagnosis for both amyloidosis groups was bilateral carpal tunnel syndrome (ATTRwt amyloidosis: 56%; ATTRv amyloidosis: 30%). Respondents with ATTRwt amyloidosis most commonly reported being diagnosed with thickened left ventricular heart wall (70%), followed by atrial fibrillation (48%) and sleep apnoea (44%), prior to formal diagnosis. For those with ATTRv amyloidosis, common diagnoses prior to diagnosis with amyloidosis included thickened left ventricular wall, rotator cuff tendon tear, trigger finger and irritable bowel syndrome (20% for each).

#### Procedures during journey to diagnosis

Survey respondents with ATTRwt amyloidosis underwent substantially more procedures to manage their symptoms and reach a correct final diagnosis, compared with patients with ATTRv amyloidosis (31% of patients with ATTRwt reported experiencing ≥ 4 procedures prior to diagnosis of wtATTR amyloidosis versus 5% patients with ATTRv). Orthopaedic procedures were the most prevalent across both amyloidosis types. Over 50% of respondents with ATTRwt amyloidosis required carpal tunnel release surgery in both wrists, typically 4 or more years before diagnosis. Respondents with ATTRv amyloidosis frequently underwent carpal tunnel release surgery in one wrist (20%), with it being performed most often either more than 10 years before or 0–3 years before diagnosis. Shoulder repair surgery was also a common procedure (10%) occurring 0–3 years before diagnosis.

#### HCP interactions

**Fig. 7:**
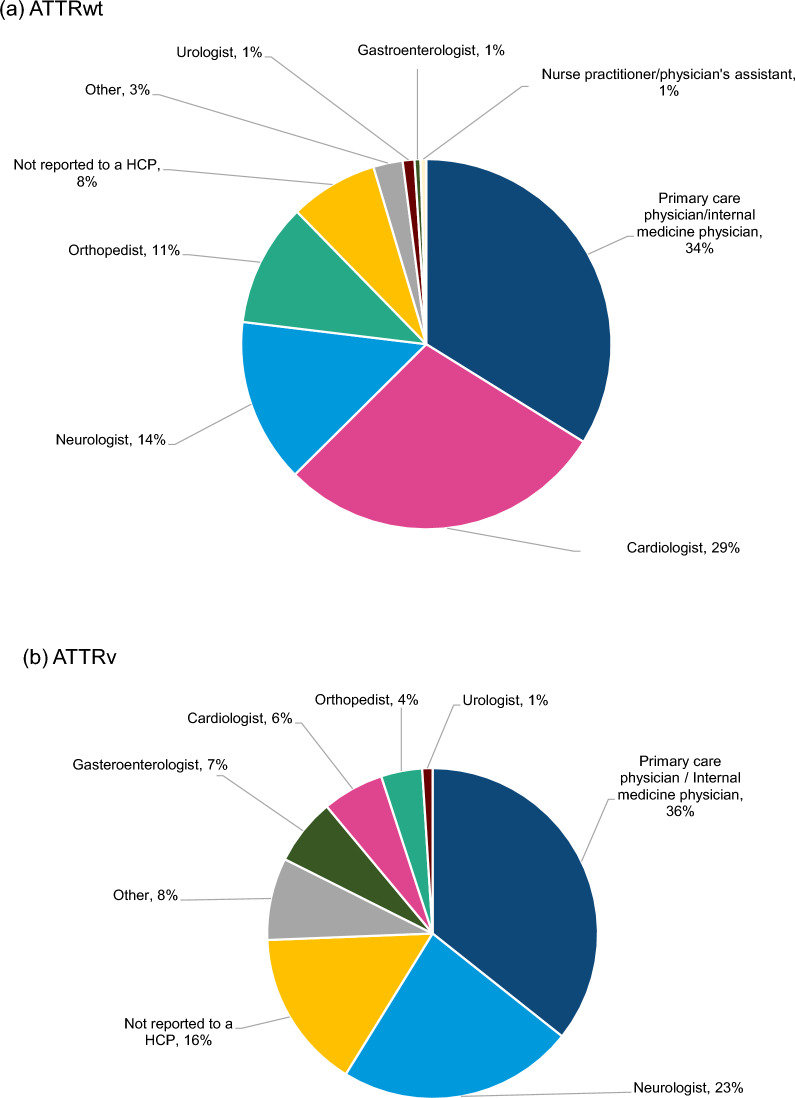
Healthcare practitioners consulted for signs and symptoms by survey respondents. ATTRv, Hereditary transthyretin; ATTRwt, Wild-type transthyretin; HCP, Healthcare practitioner. *Notes*: Healthcare practitioners consulted for signs and symptoms by survey respondents with **a** ATTRwt amyloidosis (n = 26) and **b** ATTRv amyloidosis (n = 18)

Survey respondents with either type of amyloidosis reported seeing a mix of HCPs during their journey to diagnosis. These were mainly primary care physicians, neurologists, cardiologists, and orthopaedists, owing to the variety of symptoms they experienced (Fig. 7a, b). Before their ATTR amyloidosis diagnosis, survey respondents with ATTRv and ATTRwt amyloidosis primarily visited cardiologists, followed by orthopaedists. Cardiac signs and symptoms reported by respondents with ATTRwt amyloidosis were atrial fibrillation, atrial flutter, or atrial tachycardia and thickened left ventricular wall. Cardiac signs and symptoms reported by respondents with ATTRv amyloidosis were thickened left ventricular heart wall and atrioventricular block. Orthopaedic signs and symptoms reported by respondents with ATTRwt amyloidosis were bilateral carpal tunnel syndrome and biceps tendon rupture. Orthopaedic signs and symptoms reported by respondents with ATTRv amyloidosis were rotator cuff tendon tear and bilateral carpal tunnel syndrome in both wrists.

Respondents with ATTRwt amyloidosis primarily received other diagnoses from orthopaedists and underwent a greater number of procedures (mainly orthopaedic) compared with respondents with ATTRv amyloidosis. The latter respondents most often received other diagnoses from cardiologists and underwent fewer procedures before formal diagnosis. The second-most-common type of HCP to whom respondents reported their signs and symptoms differed between the two subgroups. While a large proportion of respondents with ATTRwt amyloidosis reported their signs and symptoms (e.g., shortness of breath, heart palpitations/heart fluttering and swelling in lower legs/feet) to a cardiologist, those with ATTRv amyloidosis often reported their signs and symptoms (e.g., fatigue, tingling in feet and numbness in feet) to a neurologist.

### Interview results

Out of the 14 participants with ATTRwt interviewed, the diagnostic journey spanned a median of 16 years. Some participants were diagnosed within 3 years of experiencing their first suspected amyloidosis-related symptom, while one participant reported their first suspected amyloidosis-related symptom 32 years prior to their diagnosis. Irrespective of the length of the diagnostic journey, participants reported that the process was physically burdensome, frustrating, and emotionally taxing.

#### Experience of symptoms of ATTRwt amyloidosis

Participants consistently reiterated that ATTRwt amyloidosis is a multisystem, heterogeneous disease with wide-ranging impacts on all aspects of their lives. The most prevalent symptoms reported by interview participants mirrored those found in the survey. During the early phase of the diagnostic journey, steadily worsening physical health was the most reported sign of disease progression. Twelve out of 14 participants noted being unable to physically exert themselves in the same way as previously, or having a decline in their ability to perform tasks (see participant quotes in Table 3).

**Table 3:** Incremental changes reported during participant interviews

Incremental changes in physical health	
Participant 1	“I was not as physically adept as I used to be”
Participant 2	“…it was a progressive lessening of my ability to perform physical activities”
Participant 6	“I was having a lot more trouble breathing, especially on exertion. So, it was just steadily getting worse”
Participant 8	“…over a period of time I noticed that I was less able to [ride my mountain bike, ride our horse, play basketball, hike]”
Incremental changes in neuropathy-related and cardiovascular-related manifestations	
Participant 3	“I’m on these walks where I’ll get out of breath within a minute or two or three and have to stand there for 5, 10 s. I’ll walk a little ways further, stop, walk again”
Participant 6	“I had another incident where I was getting off the road to take a nap, because I knew I was going to fall asleep, and I fell asleep before I got to the parking space… And, so, I knew something was wrong. I was always tired”
Participant 6	“My breathing got just absolutely horrible. I was completely out of breath while sitting. I couldn’t even talk. Then, over time, it just made it harder and harder for me to breathe until I really, (my wife) and I thought I was going to die”
Participant 7	“Eventually, I developed that arrhythmia… And that was somewhat periodic. But that’s what led to that pacemaker being inserted into my body when I was hospitalized”
Participant11	“The biggest issue for me is, is the cardiac… before, I could snow ski, I like to sail. Those are things that I am hesitant to do now because I just don’t have the capacity to breathe”
Incremental changes in orthopaedic-related manifestations	
Participant 6	“It would happen at night, and mostly, between my hip, and trigger fingers, and all the other orthopaedic problems, I lost a lot of sleep. I have basically been sleep-deprived since forever. And getting some resolution to the trigger fingers was necessary because they had just gotten so bad that I couldn’t function”
Participant 1	“I think for me the key is my orthopaedic situation, my [erectile dysfunction] situation, and my stamina. Those are the key things that, I will call it for my enjoyment of life”
Participant 12	“I would wake up literally screaming because of the pain in my legs. At some point, that pain also moved to my arms and hands, kind of came up my legs. Nobody could figure out what it was”
Participant 3	“Shortness of breath, muscle weakness. All of it is part of the package, it’s just something that you have to deal with”
Participant 9	“I’m having problems with leg cramps when my legs are up that way due to the neuromuscular stuff, due to the neuropathy. I’m spending most of the nights in a recliner in the bedroom…I’m unsteady on my feet, so I have to use a cane to get around”
Participant 13	“I would guess probably the sleep apnoea (impacted me the most), because it was the one that was most ongoing”

Manageable, incremental changes in health status were followed by more impactful manifestations, the effects of which were felt in various spheres of participants’ daily lives. Palpitations, shortness of breath, and fatigue were some of the most prevalent participant-reported symptoms. These had a substantial effect on participants’ mobility and physical health, and severely affected their ability to conduct daily activities (Table 3).

Among orthopaedic manifestations, carpal tunnel syndrome was the most reported sign in the population of interviewees with ATTRwt amyloidosis (n = 9 of 14), followed by trigger finger (n = 4 of 14). The associated pain led to significant sleep disturbances, which itself impacted physical and mental functioning during the day (Table 3).

Pain and erectile dysfunction were also commonly reported signs. As outlined in the Methods section, manifestations were pre-categorized into three categories—orthopaedic, cardiovascular, and other (not cardiovascular or orthopaedic)—to provide an analysis scheme for the report (note that these categories are not clinically validated aetiological groups, and certain symptoms are multifactorial). Pain and sleep apnoea had the largest impact on participants’ physical functioning, while erectile dysfunction had the largest impact on the participants’ relationships as it affected their intimacy with their partners (Table 3).

Sleep apnoea and musculoskeletal pain, which were previously discussed in the survey, were also reported by participants as having a large impact on their physical functioning and daily lives. The cumulative effect of all manifestations presented an important burden and impediment on the participants to live their lives (Table 3).

#### Journey towards diagnosis of ATTRwt amyloidosis

Owing to the length of the journey and the multiplicity of manifestations experienced by participants, most reported being referred to a range of different clinical specialists and undergoing numerous tests and procedures before being diagnosed (see selection of participant quotes in Table 4).

**Table 4:** Participant journey toward diagnosis of ATTRwt amyloidosis reported during participant interviews

Length of journey	
Participant 6	“I had a cervical fusion, and lumbar release, and one cubital tunnel release, all at about the same time. And also, in the middle of that, I’m still having a lot of trouble breathing”
Participant 10	“My cardiologist did many, tests over quite a period of time, maybe 2009 to maybe 2014, about 5 years, did many tests and then that’s when he finally did an MRI of the heart”
Participant 14	“We probably forget how stressful it all was because it was like there were appointments for one thing or another, neurology, oncology, cardiology, family practice, it was a very, busy time for about two and a half years. And then it was very stressful until we had to wait about a month to get to [the centre of excellence]”
HCP interactions	
Participant 11	“…all the different doctors, the cardiologist, the haematologist, oncologist, just nobody was coordinating, didn’t seem like anybody was coordinating the treatment. You had to kind of pick and choose what they were telling you and kind of do it on your own”
Participant 14	“We really started to worry about thinking we may have been on our own path”
Participant 12	“I went to first an orthopaedic and then they sent me to a neurologist and they sent me to a pain specialist and they sent me back to orthopaedic. Around and around we went until somebody finally sent me to the spine centre”
Participant 12	“No one talks to one another and it’s very frustrating because then I end up telling everything all the way over again. They have access to all my records of course, but there’s no soft hand-off. It’s like, ‘Okay, you’re not my problem anymore. Move on.’ That’s what it feels like”
Health insurance perspective	
Participant 11	“So this was all new to me. I mean, the process of insurance, coordinating with different doctors is a nightmare for somebody that’s already sick. […] But we had a heck of a time getting on the same page with the insurance company and then all the different doctors…”
Health insurance implications	
Participant 7	And then I was hospitalized on January of 2016 at not the best hospital, not the one that I was involved with, but the closest one. […] About a year and a half ago when I saw him, I had to fly to [academic centre] twice a year”
Participant 11	“So it’s a real struggle, especially because I had that advantage plan where you couldn’t go out of network without paying. I ended up paying about $5,000 just to go to [academic centre] or more, because…my wife and my son came with me as well”

Participants reported that they felt that the many different physicians and the high number of procedures resulted in a high burden of manifestations, symptom progression, and misdiagnoses (theme: feeling like ‘a lab rat’). Participant 3 described the process as “this never-ending situation of more doctors’ appointments, changes in medication, a-fib, sinus rhythm”. They kept a record of their journey and, during the interview, referred to their notes pointing to a “four-page document of every doctor’s appointment I had going back to when I first started noticing heart issues, shortness of breath, dizziness. So, it’s just a chronological record of all the people that I saw, all the different medications they put me on, all the ablations, all the cardioversions, before they finally recognized what was going on and sent me to (centre with experience managing amyloidosis redacted) critical heart care specialist and I got this thing all figured out”. Participant 12, who saw approximately eight different doctors before diagnosis, described the situation succinctly: “It was frustrating. I did feel a little bit maybe like a volleyball being bounced over from one to another to another.”

A key issue was that the medical specialists that participants saw often worked in unintegrated silos, and participants felt that they were burdened with the responsibility of communicating across an array of HCPs (Table 4).

On top of navigating various medical specialists, participants reported that coordinating care was also particularly challenging from a health insurance perspective. While most participants interviewed felt they had good coverage, this may not be representative of the wider population with ATTR amyloidosis (Table 4).

Examples of insurance implications for participants included navigating: coverage for out-of-network ATTR amyloidosis specialists; changes in coverage after reaching retirement age; and eligibility for Medicare. Participant 9, for example, mentioned that transitioning to Medicare involved a “whole new learning curve”; however, this was manageable and had little impact on the participant’s life. In contrast, out-of-pocket costs for certain participants were significant. Participants 7 and 11 reported having to travel out of state to access specialist care before their diagnosis (Table 4).

#### Key indicators of ATTRwt amyloidosis

Interview participants were typically diagnosed only after the disease had progressed to include severe cardiac symptoms such as atrial fibrillation and severe shortness of breath. Atrial fibrillation and other cardiovascular-related events (e.g., thickened left-ventricular wall) were important triggers that brought participants closer to a formal diagnosis. However, musculoskeletal symptoms (particularly carpal tunnel syndrome and trigger finger) were very prevalent. Participants believed these musculoskeletal symptoms were often overlooked and could be considered missed opportunities for diagnosis by HCPs. Specifically, just over half of participants singled out carpal tunnel syndrome as a key missed opportunity for an earlier diagnosis (Table 5).

**Table 5:** Key indicators of ATTRwt amyloidosis reported during participant interviews

Carpal tunnel syndrome	
Participant 2	“I guess the only one that comes out at me is that bilateral carpal tunnel going back harder and pushing forward harder, doing some of my research, but I don’t know about that would have gotten connected into the cardiology part of it through my looking around perhaps something would have popped up but that would be the only one”
Participant 7	“But in 2012, when I was [age] I saw a hand surgeon, I asked him, when he did the surgery on my right wrist, to do a biopsy of the transverse ligament to look for amyloidosis. And, of course, he neglected to do that. So, I could, in theory, have been diagnosed in 2012 as opposed to 4 years later”
Participant 8	“Going back to when I was [age] and when I had the carpal tunnel surgical release, if at that time that hand surgeon had consulted or presented the material that he removed from my carpal tunnel to a pathologist or had a very simple test done for that material, a Congo red stain, that would’ve disclosed amyloidosis at that time”
Participant 10	“I guess if we did the carpal tunnel surgery, we did the knee surgery, and we did the rotator cuff surgery, and samples had been taken of my tissue at that point and tested for ATTR, that might’ve helped”
Participant 11	“If I had gone in on my own just to have somebody look at my carpal tunnel, my hands, I could’ve—but other than that, I don’t know of any—I mean, just snuck up on me”

## Discussion

In this mixed-methods study involving both quantitative and qualitative data from people with ATTR amyloidosis, the findings demonstrate that the symptomatic burden of disease and diagnostic journey leading up to the diagnosis of ATTRwt and ATTRv amyloidosis were exhausting for patients. The responses and interviews show that patients experience progressive signs and symptoms across the musculoskeletal, cardiac, and peripheral nervous systems, revealing numerous opportunities to intervene years before formal diagnosis. These findings underscore previous research examining the diagnostic journey and burden of illness of ATTRv amyloidosis. Crucially, they also provide patients’ perspectives regarding the impact of seeing different HCPs, and the early manifestations of ATTRwt amyloidosis on patients’ daily functioning, quality of life, and relationships.

People with ATTR amyloidosis saw several different types of HCPs for years leading up to their formal diagnosis to manage their various signs and symptoms. Feedback indicated that HCPs often worked in silos with suboptimal communication and a general lack of awareness for ATTR amyloidosis. This lack of HCP coordination resulted in many participants undergoing additional assessments, referrals and procedures, which increases healthcare resource utilization. This, in combination with their deteriorating health, meant that participants often reported being fearful, anxious, and frustrated at feeling the need to be their own advocates within the process.

Musculoskeletal signs and symptoms, particularly carpal tunnel syndrome and trigger finger, were prevalent in over 50% of participants with ATTRwt amyloidosis up to 10 years before formal diagnosis. Recent publications noted how musculoskeletal manifestations are prominent early indicators of ATTRwt amyloidosis and that they often precede the cardiac symptoms common to the condition [31, 32]. Carpal tunnel syndrome could be an important indicator of ATTRwt amyloidosis that offers opportunities for early detection and diagnosis [32]. Participants identified these signs and symptoms as early risk identifiers, but that they were missed by HCPs. Since conducting the interviews, two further studies support the use of carpal tunnel syndrome as a red flag that precedes diagnosis [19, 20]. In their study, Ladefoged et al. screened patients aged over 59 with a recent history of carpal tunnel syndrome for ATTRwt amyloidosis, and found that 8.3% of patients had ATTRwt amyloidosis [20]. Similarly, Brunet et al. found that, of 254 patients aged between 60 and 80 undergoing carpal tunnel decompression, 18.5% had transthyretin amyloidosis [19].

Increasing awareness among HCPs involved in the earlier stages and manifestations of ATTRwt amyloidosis, and promoting a multidisciplinary approach to patient care coordination, would support the earlier recognition of signs and symptoms of ATTRwt amyloidosis and timelier diagnosis. Furthermore, as many patients with ATTRwt amyloidosis require musculoskeletal procedures (e.g., spinal stenosis, rotator cuff tear, and biceps tendon rupture) early in their journey to diagnosis, orthopaedists are important stakeholders to include in multidisciplinary discussions relating to patients' medical history and care.

There are currently high levels of uncertainty regarding whether sleep apnoea is an early sign or symptom of ATTRwt amyloidosis. It was experienced by 44% of respondents with ATTRwt amyloidosis prior to their formal diagnosis and was reported in the interviews as having the largest impact on interview participants’ physical functioning (along with pain). It is unclear whether sleep apnoea can be considered an early diagnostic trigger for ATTR amyloidosis or simply a co-morbidity associated with heart failure. Indeed, prior studies have shown an association between obstructive sleep apnoea and heart failure [33–35]. Our work suggests sleep apnoea as a symptom that requires further investigation in ATTR amyloidosis.

Other key indicators of ATTRwt amyloidosis included atrial fibrillation and thickened ventricular wall, which aligns with the known role of cardiomyopathy in this disease. Of note, a recent systematic literature review on the screening and diagnosis of wild-type transthyretin amyloid cardiomyopathy found that the most frequently mentioned suspicion criteria were ventricular wall thickening [36]. Unsurprisingly, all but one of the participants interviewed were diagnosed by their cardiologists, confirming the typical diagnostic pathways of patients with ATTRwt amyloidosis.

Fatigue was reported as a particularly burdensome symptom by both participants with ATTRv amyloidosis and ATTRwt amyloidosis. Understanding the role of chronic fatigue and its clinical significance in ATTR amyloidosis is challenging. It has a complex aetiology, as it can itself be caused by other symptoms of ATTR amyloidosis (for example, due to pain-related sleep disturbance, or as a result of cardiac-related symptoms) as well as other unrelated conditions. Still, our results align with those of a 2016 US Food and Drug Administration report summarizing perspectives of people with amyloidosis, in which patients identified fatigue as a significantly debilitating symptom [37].

Two previous studies (Damy et al., 2022; Ladefoged et al., 2020) highlighted that a diagnosis of ATTRwt amyloidosis was delayed, occurring 34.4 [1] and 13 months [38], respectively, after the onset of polyneuropathy and/or cardiomyopathy symptoms. Our study shows that when orthopaedic signs and symptoms are included as part of the diagnostic journey for ATTRwt amyloidosis, the journey to diagnosis could be considered much longer—up to 16 years in some cases. This highlights an increasing complexity of a patient’s case, as they acquire new issues with mobility, dexterity, pain, numbness, and experience numerous surgeries over time.

Detecting and diagnosing ATTR amyloidosis is complicated by the fact that some symptoms indicative of the disease reported in our study are also highly prevalent in the population without amyloidosis. Thus, the individual occurrence of a potential amyloidosis-related symptom, or even co-occurrence of multiple symptoms, does not initially suggest an aetiological relationship with amyloidosis. For example, symptoms of chronic fatigue may simply be due to ageing. However, the perspectives given by the participants show how the accumulation of such symptoms over time and their severity serve as indicators of amyloidosis—and as key drivers of their continued engagement with HCPs across different specialties. Our retrospective analysis illustrates how the story can be ‘pieced together’ with the benefit of hindsight, but also provides learnings for how the patient experience can be improved throughout the diagnostic journey.

Patients with ATTRwt amyloidosis underwent substantially more procedures to manage their symptoms and reach a correct final diagnosis, compared with patients with ATTRv amyloidosis. This suggests a higher level of healthcare resource use for people with ATTRwt amyloidosis. Recognizing early signs and symptoms can potentially reduce the burden of multi-year diagnostic journeys on healthcare systems for both ATTRv and ATTRwt amyloidosis—especially the latter. Our study further highlights the impact on the healthcare system of ATTRwt amyloidosis relative to ATTRv amyloidosis prior to diagnosis, an effect that has not been investigated in this population.

Timely diagnosis of ATTRwt amyloidosis is a clear unmet need and should be met with pragmatic solutions. To aid HCPs’ ability to recognize this condition, approaches should be taken that aim to: 1) strengthen inter-specialty coordination; and 2) map the myriad of symptoms present with ATTRwt amyloidosis that could occur alongside more common conditions. For example, while early symptoms such as carpal tunnel syndrome are common problems among the general population, it may be worth measuring how many patients experience additional cardiomyopathy- and/or neuropathy-related issues and go on to develop ATTRwt amyloidosis later in life [39]. If early signs and symptoms could be confirmed and agreed upon within clinical practice, then well-defined multidisciplinary care models and guidance could be developed. Modern technical applications, including learning algorithms and electronic medical records data mining/scoring systems to find patients most at risk, could be utilized to create such guidance. This could then be shared between local and expert care to allow for enhanced coordination in the steps towards ATTRwt amyloidosis diagnosis.

To date, a number of diagnostic algorithms and clinical recommendations based on expert consensus have been published to take stock of early signs of the ATTRwt amyloidosis [40–43]. Since performing interviews, pilot studies have been conducted to incorporate predictive analytics into the electronic health records of academic medical centres to identify at-risk patients. In their study, Willis et al. adapted an early risk prediction model to help identify patients with heart failure that may be at risk of ATTRwt, a tool that may guide other centres to implement similar systems to aid screening and diagnosis [44].

Educating HCPs on the most common and early signs of ATTRwt amyloidosis and fostering prompt referral to centres with experience could also help reduce the diagnostic delay. Interviewing HCPs about their experience with and exposure to ATTR amyloidosis could therefore provide insight into diagnostic challenges from the clinician’s perspective.

Two key strengths of our study were the approach taken and the use of qualitative interviews. Our mixed-methods approach allowed us to gather enough information to recognize patterns in the signs and symptoms of ATTRwt amyloidosis, as well as learn about the participants’ diagnostic journeys and the type and number of HCPs involved in their care and diagnosis. The qualitative interviews enabled us to explore the survey findings in depth and hear the first-hand experiences of people living with ATTRwt amyloidosis. The interviews also highlighted key areas of unmet need and provided potential insight (e.g., early symptoms and key HCPs involved in the care coordination) that could form the basis for a medical strategy to improve the diagnostic journey of patients with ATTRwt amyloidosis.

Sample representation and sample interview size were limitations of this study. All participants were members of the ASG and may represent a more informed and better resourced group than the generalizable patient population. While the symptomatic and diagnostic burden in this group was evident, the findings of this research may be biased towards representing a ‘best-case scenario’ in their reflection of the diagnostic journey. Patients with ATTRwt amyloidosis from disadvantaged socio-economic backgrounds without a strong network, medical connections, or financial resources may struggle even more to find the right information, get a timely diagnosis, and receive appropriate care. Among survey respondents, there was a low representation of people with the mutation *TTR* V122I, the most common mutation in the US and one that predominately impacts African Americans [45]. Research has shown that the journey to diagnosis is longer in this patient population and that they generally have poorer prognoses [16].

Since symptoms were patient reported, the extent to which they were directly attributable to the disease is uncertain. As such, it is not possible to ascertain the full aetiology of the manifestations reported as part of the survey results and interviews. In addition, the qualitative interviews focused on extracting the impact that symptoms, procedures, and other events before ATTRwt amyloidosis diagnosis had on participants’ lives. This journey spanned decades for many participants, so recall bias was an important study limitation. To mitigate this issue, many participants used notes of their medical records. In three interviews, the participant’s partner took part in the discussion, providing additional insight to piece together the timing of the different events of their diagnostic journey.

Because the interviews were only conducted with participants with ATTRwt amyloidosis, the burden reported in the in-depth interviews cannot be directly comparable with that found in the literature for patients with ATTRv amyloidosis. Additionally, age may be a confounding factor for some of the differences identified between participants with ATTRv and ATTRwt amyloidosis.

## Conclusion

This study provides a better understanding of the burdensome, frustrating and emotionally taxing diagnostic journey experienced by people with ATTRwt amyloidosis. Our results demonstrate that there are opportunities for earlier diagnosis, especially if HCP awareness and coordination improve, and early indicators such as carpal tunnel syndrome are used as the basis for ATTRwt screening. New potential indicators, such as sleep apnoea, could also serve as additional pre-cardiac marker of the disease if their correlation with ATTRwt is further confirmed. In turn, earlier recognition of the condition would aid in the management of the high diagnostic and symptomatic burden that impacts every aspect of patient’s lives, and provide opportunities for earlier treatment.

## Supplementary Information

Additional file 1: Study protocol.

Additional file 2: Interview guide.

Additional file 3: Patient journal map template.

Additional file 4: List of procedures and symptoms given to participants.

## Data Availability

The authors confirm that the data supporting the findings of this study are available within the article [and/or] its supplementary materials. For further information, the corresponding author may be contacted.
