# Supplementary material for: The journey to diagnosis of wild-type transthyretin-mediated (ATTRwt) amyloidosis: a path with multisystem involvement
Source: Orphanet J Rare Dis. 2024 Nov 8;19:419. doi: 10.1186/s13023-024-03407-3 (PMC11549766; doi:10.1186/s13023-024-03407-3)
Supplement: Supplementary file 1 — Additional file 1: Study protocol. [file 13023_2024_3407_MOESM1_ESM.pdf]

Transthyretin Amyloidosis Patient Survey: Evaluating the Journey to Diagnosis in Hereditary and Wild-type Transthyretin-Mediated Amyloidosis

Protocol version 5.0

Report date:

5 May 2022

Contents

1. Overview study details ... 4
2. Introduction ... 7
   - 2.1 Background information ... 7
   - 2.2 Study Rationale ... 8
   - 2.3 Research objectives ... 9
   - 2.4 Study design ... 10
     - 2.4.1 Study background ... 10
     - 2.4.2 Recruitment process ... 11
     - 2.4.3 Sample size ... 11
     - 2.4.4 Informed consent ... 12
3. Methodology ... 12
   - 3.1 Part 1: Survey administration ... 12
     - 3.1.1 Survey selection criteria ... 12
   - 3.2 Part 2: Interview administration ... 13
     - 3.2.1 Interview selection criteria ... 13
     - 3.2.2 Interview guide design ... 13
     - 3.2.3 Interview process ... 14
   - 3.3 Data collection and data storage ... 14
   - 3.4 Data analysis and reporting ... 15
     - 3.4.1 Analysis ... 15
     - 3.4.2 Reporting ... 17
   - 3.5 Confidentiality and data protection ... 18
     - 3.5.1 Confidentiality and data protection ... 18
     - 3.5.2 Adverse event reporting ... 19
4. Ethical review ... 20
   - 4.1 Reimbursement and compensation for research participants ... 20
5. References ... 21

Abbreviations

Abbreviation	Definition
ASG	Amyloidosis Support Groups
ATTR	Transthyretin-mediated amyloidosis
FAC	Familial amyloid cardiomyopathy
FAP	Familial amyloid polyneuropathy
GI	Gastrointestinal
ATTRv amyloidosis	Hereditary transthyretin-mediated amyloidosis
HCP	Health care professional
QOL	Quality of life
TTR	Transthyretin-mediated
ATTRwt amyloidosis	Wild-type transthyretin-mediated amyloidosis

1. Overview study details

Table 1: Overview of study details

Overview of amendments to the ethics protocol	
**Characteristic**	**Description**
**Study history**	• Ethics approval granted November 24, 2020 to conduct a one-time online survey with US patients with ATTRv and ATTRwt amyloidosis<br>• Enrolment for the survey was between November 30–December 8, 2020<br>• Amendment #1: The survey received 1,022 ‘clicks’ into the survey, due to this unexpected high number of responses, the aim of the first ethics amendment was to obtain approval for an additional validation step to identify ‘honest’ responders<br>○ This step included a cross-check of survey respondent email addresses with the ASG patient association membership list<br>○ This process received ethics approval on November 10, 2021.<br>• Amendment #2: Conduct of qualitative interviews with a subset of participants from the online survey
**Study Overview**	
**Characteristic**	**Description**
**Study type**	Part 1: Online, patient-completed survey<br>Part 2: Qualitative patient interviews
**Research objectives**	**Part 1 – Survey:**<br>Primary objectives:<br>• Understand the disease presentation (diagnoses, signs/symptoms, procedures) between the time of the first suspected symptom and date of diagnosis in patients with ATTRv or ATTRwt amyloidosis<br>• Evaluate healthcare provider interactions between first suspected symptom and date of diagnosis<br><br>Secondary objective:<br>• Evaluate the similarities and differences in the diagnostic journeys of ATTRwt and ATTRv amyloidosis patients<br><br>**Part 2 – Interviews:**<br>• Gain an in-depth understanding of the overall disease burden and symptom development during the diagnostic journey from the patient’s perspective, including the impact on quality of life (QOL), ability to work, relationships, and general health status<br>• Assess the key milestones, including interactions with healthcare providers and major disease worsening events, and the timeline of early disease progression from the patient perspective<br>• Evaluate the quality of the patients’ interactions with the healthcare system during their diagnostic journey, assessing both drivers that assisted in making the diagnosis of ATTR amyloidosis, as well as unmet medical needs during this time

**Population**	Part 1 – Survey: US adult patients with ATTRv or ATTRwt amyloidosis<br>Part 2 – Interviews: Initially, interviews will be conducted in ATTRwt amyloidosis patients who have been diagnosed with cardiomyopathy; however, interviews may be expanded to include any ATTR amyloidosis patient
**Planned number of participants**	Part 1: Aim was to enroll a minimum of 60 patients (~30 patients with ATTRv amyloidosis and ~30 patients with ATTRwt amyloidosis); however, there was no cap on patient participation from either group and enrollment depended on patient interest and eligibility<br><br>Part 2: Aim is to reach data saturation (point where no new information is being shared), which is anticipated to be met after around 15 patient interviews. If saturation is not reached or the investigators wish to expand the interview population, approximately 10 additional interviews may be conducted. If saturation is not met after 25 interviews, the population may be subsequently expanded.
**Collaborating parties involved in the design and recruitment of the survey**	• Alnylam Pharmaceuticals, Inc research team (Alnylam)<br>• ATTR amyloidosis patients including members of the Standing Patient Advisory Board*<br>• ASG leadership (Muriel Finkel, Paula Schmitt)<br>• Clinical experts**: Drs. Mazen Hanna and Chafic Karam<br>• BresMed
**Inclusion criteria**	• Patients aged 18 and over<br>• Patients with a confirmed diagnosis of ATTRv or ATTRwt amyloidosis<br>• English language<br>• Patient resides in the US<br><br>Part 2 – Interviews:<br>• Completed Part 1<br>• Able and willing to participate in a virtual or telephone interview
**Methods**	Part 1 – Survey: Web-based survey (including screener questions) that presents predominantly closed-ended questions with a minority of open-ended questions to elicit both qualitative and quantitative responses<br><br>Part 2 - Interviews: Patients will be contacted for a 1-1.5 hr telephone interview; after providing verbal or written consent to participate, patients will respond to open-ended questions posed by the researcher based on a finalized interview guide. Interviews will be recorded and transcribed for analysis purposes.
**Analysis**	Part 1 – Survey:<br>• Only data from those survey participants whom fit the inclusion criteria and 1) the ASG confirms are patients living with ATTR amyloidosis or 2) completed the survey but did not request financial compensation for their time and did not disclose their email address were included in the analysis (See section 3.3)<br>• Descriptive statistical analysis were performed on the closed-ended questions. In addition, thematic analysis will be conducted on the qualitative data generated by open-ended responses<br><br>Part 2 - Interviews:

	• Transcripts from the quantitative interviews will be thematically coded. Qualitative thematic / framework analysis using Nvivo Software will be undertaken.
**Key:** ASG, Amyloidosis Support Group; ATTR, Transthyretin-mediated amyloidosis; ATTRv, hereditary transthyretin-mediated amyloidosis; HCP, healthcare professional; US, United States; ATTRwt, wild-type transthyretin-mediated amyloidosis.<br>**Notes:** *Patients on the Standing Advisory Board are compensated by Alnylam for their time. ** Clinical experts are compensated by Alnylam for their time in accordance to the Sunshine act / California law at fair market value.	

2. Introduction

2.1 Background information

Transthyretin-mediated amyloidosis (ATTR) is a rare, underdiagnosed, progressive, debilitating and fatal disease. It is caused by misfolded transthyretin (TTR) proteins that accumulate as amyloid deposits in multiple tissues, including heart, nerves and the gastrointestinal (GI) tract. ATTR encompasses two disease types: hereditary transthyretin-mediated (ATTRv) amyloidosis, also known as hATTR amyloidosis, and wild-type transthyretin-mediated (ATTRwt) amyloidosis, also known as wtATTR amyloidosis.

ATTRv amyloidosis is a multisystem disease with a heterogeneous clinical presentation that includes sensory and motor neuropathy, autonomic impairment (e.g., diarrhea, sexual dysfunction, orthostatic intolerance), and cardiac signs and symptoms. The majority of patients with ATTRv amyloidosis develop a mixed phenotype of both polyneuropathy and cardiomyopathy.[1-7] ATTRv amyloidosis has an aggressive course with rapid disease progression leading to deteriorating quality of life (QOL), loss of function, and a median survival of 4.7 years following diagnosis, with a reduced survival (3.4 years) for patients presenting with cardiomyopathy.[1, 7-12]

ATTRwt amyloidosis is a fatal disease caused by the accumulation of misfolded TTR occurring predominantly in the heart although multisystem involvement has also been reported.[1, 3, 6, 8, 13-15] A proportion of patients with ATTRwt amyloidosis experience sensory and motor neuropathy and autonomic impairment (e.g., GI symptoms, orthostatic intolerance). Orthopedic manifestations (e.g., carpal tunnel syndrome, lumbar spinal stenosis, biceps tendon rupture) have been reported.[14, 16] ATTRwt cardiac amyloidosis is a progressive disease leading to deteriorating QOL and loss of function, with natural history studies showing a median survival of 2.5–5.5 years following diagnosis.[17-23]

Epidemiology

ATTRv amyloidosis represents a major unmet need with significant morbidity and mortality, affecting approximately 50,000 people worldwide.[2, 24] The number of people with ATTRv amyloidosis in the US is estimated to be 26,000–37,000 but only 10–30% are estimated to have been diagnosed.[25] Although limited epidemiology data are available for ATTRwt amyloidosis, estimates suggest there are 2000–115,000 patients in the US.[15, 26, 27]

Diagnosis

There has been an increase in the variety of diagnostic tools available following initial suspicion of ATTR amyloidosis. This increase highlights the importance of early diagnosis due to the rapidly progressive, fatal nature of the disease and the need for early therapeutic intervention to support the best possible outcome for patients.[28] However, patients often experience delays in their

diagnostic journey due to non-specific initial symptoms and low disease awareness in clinical practice.[29]

The average age of diagnosis of ATTRwt amyloidosis is 75 years; however, missed or delayed diagnosis is common as many healthcare professionals (HCPs) are unfamiliar with amyloidosis and may assume the patient's heart problems are associated with complications of older age and more common chronic diseases.[14, 28, 30-32] Yet, there is a suggestion that patients have common symptoms, for example 50% of patients with wild-type experience carpal tunnel syndrome, often 3–5 years before symptoms of heart disease.[33]

2.2 Study Rationale

To date, limited data is available pertaining to the clinical characteristics, prevalence, and burden of early ATTR amyloidosis-related disease manifestations in US patients. In particular, the early disease course of ATTRwt amyloidosis (i.e., clinical features occurring prior to heart failure) are not well characterized. The literature indicates that patients may experience neuropathy and orthopedic complications years in advance of severe cardiac dysfunction in both ATTRv and ATTRwt amyloidosis, but little has been reported on the prevalence, timing, and severity of such non-cardiac symptoms. Additionally, two clinical syndromes of ATTRv amyloidosis have been described in the medical literature: familial amyloid polyneuropathy [FAP] or familial amyloid cardiomyopathy [FAC].[34] This characterization is due in part to the incomplete understanding of etiology and pathogenesis in hereditary amyloidosis. More recent literature has reported that most patients with ATTRv amyloidosis manifest signs and symptoms of both polyneuropathy and cardiomyopathy over the course of their disease.[5-7, 35, 36] Finally, due to the genetic nature of ATTRv amyloidosis, the time between early disease symptoms and diagnosis may differ in patients with a known family history compared to those who are index cases.

This observational, patient-completed online survey study with follow-on, in-depth qualitative interviews aims to achieve a better understanding of the natural history of ATTRv and ATTRwt amyloidosis leading up to diagnosis and the impact of this period of the disease on patients' lives. Relevant medical history, including signs, symptoms, diagnoses and procedures, that have been reported in the literature or reported by patients or physicians to be associated with ATTR amyloidosis were assessed. In addition, the timing of each manifestation and the physician specialty to whom these early manifestations were reported or by whom these early manifestations were managed were collected in order to gain a better understanding of the patient journey through the healthcare system during this time.

In the next phase of the research, patients will participate in qualitative interviews to provide in-depth reports of the physical, mental, and social burdens during this early stage of disease. Patients will also report key encounters with the healthcare system that aided or hindered their diagnosis. These reports are meant to further contextualize the early natural history of ATTR

amyloidosis and provide a clearer picture of how patients navigate and are impacted by the healthcare system throughout their diagnostic journeys.

This study aims to advance scientific understanding on these topics by assessing the prevalence of each clinical feature (sign, symptom, diagnosis, etc.), timing of each in relation to the diagnosis ATTRwt or ATTRv amyloidosis, and health and social consequences of early disease progression. Further, recommendations for clinical care improvements may be elucidated based on patients' descriptions of unmet medical needs during their diagnostic work-up for ATTR amyloidosis. Finally, the clinical features of each condition (i.e., ATTRwt or ATTRv amyloidosis) gathered during the survey and qualitative interviews will be qualitatively assessed to identify commonalities and differences in these two ATTR amyloidosis types.

2.3 Research objectives

Research objectives include:

Part 1 – Survey:

Primary objectives:

- Understand the disease presentation (diagnoses, signs/symptoms, procedures) between the time of the first suspected symptom and date of diagnosis in patients with ATTRv or ATTRwt amyloidosis
- Evaluate healthcare provider interactions between first suspected symptom and date of diagnosis

Secondary objective:

- Evaluate the similarities and differences in the diagnostic journeys of ATTRwt and ATTRv amyloidosis patients

Part 2 – Interviews:

- Gain an in-depth understanding of the overall disease burden and symptom development during the diagnostic journey from the patient's perspective, including the impact on quality of life (QOL), ability to work, relationships, and general health status
- Assess the key milestones, including interactions with healthcare providers and major disease worsening events, and the timeline of early disease progression from the patient perspective
- Evaluate the quality of the patients' interactions with the healthcare system during their diagnostic journey, assessing both drivers that assisted in making the diagnosis of ATTR amyloidosis, as well as unmet medical needs during this time

2.4 Study design

2.4.1 Study background

Part 1 of this study includes an observational, online, one-time survey was conducted between November 30–December 8, 2020, following ethics approval granted November 24, 2020.

This survey was completed by patients residing in the US with self-disclosed confirmed diagnosis of ATTRwt or ATTRv amyloidosis. The survey contained questions related to patient demographics, medical history (signs, symptoms, diagnoses, procedures related to neuropathic, orthopedic, or cardiac manifestations associated with ATTR amyloidosis and related physician specialty interactions), and the patient experience during the period prior to ATTR amyloidosis diagnosis.

The survey was developed in collaboration with patients from the Alnylam standing patient advisory board, the ASG leadership, clinical ATTR amyloidosis experts (including one cardiologist and one neurologist), BresMed, and the Alnylam research team. The survey was reviewed by these parties to improve the clarity and clinical relevance of the topics in relation to the research objectives. The survey had been included as an attachment to the IRB submission.

Patients completing the online survey reviewed and agreed to an informed consent statement prior to completing the survey.

At the end of the survey, patients were asked if they wished to participate in a future in depth interview. A sufficient number of patients agreed to participate, so an extension of this study is being undertaken (Part 2 of this research).

The survey received 1,022 ‘clicks’ into the survey. Due to this unexpected high number of responses, discussions occurred between the research team, clinicians and the ASG leadership on the likelihood of dishonest responses based on known baseline characteristics (age, gender for both cohorts). Therefore, the aim the first ethics amendment was to obtain approval for an additional validation step to identify ‘honest’ responders. This step included BresMed cross-checking survey respondent email addresses with the ASG patient association membership list (Alnylam had no access to participant email addresses).

Part 2 involves qualitative interviews with a subset of patients who completed the online survey and consented to being contacted regarding completing a follow-up in depth interview. During the interviews, patients will be provided with their individual journey map noting key dates, experienced manifestations and HCPs involved in their ATTR amyloidosis diagnosis journey. This would be generated from their survey results and used to probe key historical events and moments to support the semi-structured interview guides. Please see section 3.2 for more information on the methodology for how we intend to develop and conduct the interviews.

2.4.2 Recruitment process

For Part 1, a study invitation and electronic link to the survey was disseminated by ASG leadership to patient members. Specifically, this patient advocacy organization invited adult patients with ATTRv and ATTRwt amyloidosis to participate in the survey through their patient channels (e.g., email, Facebook, newsletter). The ASG also shared the study informational flyer and study link to its network of ATTR amyloidosis-treating healthcare providers (HCPs) via email. If these HCPs decided to share the study information with their ATTR amyloidosis patients under their care, they were asked to carry out the required ethics steps needed within their local clinical practice. General reminder emails developed by BresMed, Alnylam, and the ASG were sent via the ASG to help increase the response rates.

For Part 2, ATTRwt amyloidosis patients who participated in the online interviews and consented to being contacted for participation in qualitative interviews will be contacted via email by BresMed. If investigators wish to expand the sample population for qualitative interviews to gain further insights, ATTRv amyloidosis patients who participated in the online survey and consented to learn more about interviews may be contacted. Alnylam and the HCPs will not be involved in this process.

2.4.3 Sample size

Part 1

ATTRwt and ATTRv amyloidosis have relatively low prevalence in the US.[15, 26, 27] Due to the nature of the patient population and the desire to improve the generalizability of the results, there was no maximum sample size set for the online survey. Anyone meeting the selection criteria during the enrollment period were allowed to participate in the survey. However, the enrollment period was expected to remain open until a minimum of 60 patients were enrolled (~30 patients with ATTRv amyloidosis and ~30 patients with ATTRwt amyloidosis). The survey stayed open until the target sample size was reached, which was dependent on patient interest and eligibility.

Part 2

The objective of the qualitative interview sample size is to reach data saturation, i.e., the point at which no new themes relevant to the research objectives are being communicated by the patient respondents. Data saturation is a status determined by the expert researchers at BresMed. It is anticipated that saturation may be reached after approximately 15 interviews. However, if data saturation is not reached at this point, additional patients may be recruited, totalling up to 25 interviews. The majority of interviews will focus on patients with ATTRwt amyloidosis with cardiomyopathy, if data saturation is reached early, it is likely the wider ATTR population will be invited to interview, including patients with ATTRwt amyloidosis without cardiomyopathy or ATTRv amyloidosis.

2.4.4 Informed consent

For Part 1, prior to beginning the survey, patients were presented with full and adequate written information related to the study purpose, procedures, objectives, topics, data integrity, privacy protection, and possible risks and benefits of the study. Patients were informed of the voluntary nature of the study and their ability to discontinue participation at any point.

Patient’s e-consent was obtained prior to initiating the survey. E-consents are stored on a secure server and maintained by BresMed.

For Part 2, prior to conducting the in-depth interviews, patients will receive a written/e-copy of the informed consent statement. Additionally, the researcher will affirm each patient’s consent after reading aloud a consent statement at the beginning of the interview process. The informed consent form will be attached to the ethics submission.

3. Methodology

3.1 Part 1: Survey administration

The survey was made available for completion using an online platform disseminated by the ASG. The survey included both open and closed questions and was designed to take 30 minutes to complete. Participation is voluntary and participants were offered a $30 Amazon gift card as compensation for their time.

3.1.1 Survey selection criteria

The survey contained initial eligibility questions to ensure participants are interested in participating and meet the survey inclusion criteria via the following prompts multiple eligibility questions related to each inclusion parameter.

Only participants who provided their informed consent and met all inclusion criteria were able to proceed to complete the full survey. Those who were not eligible (e.g., do not meet the inclusion criteria) were diverted to the end of the survey where they were thanked for their time and provided with an opportunity to contact the research team in the event of questions.

In order to participate in the survey, patients were required to meet the following inclusion criteria:

- Patients age 18 and over
- Patients with a confirmed diagnosis of ATTRv or ATTRwt amyloidosis
- English language
- Patient resides in the US

3.2 Part 2: Interview administration

3.2.1 Interview selection criteria

Eligible participants of those initially approached via the web-based survey who are willing to participate in the semi-structured interview will be contacted by a BresMed researcher over the telephone or email (depending on the respondent's preference).

The following pre-defined inclusion criteria will be used to avoid the risk of selection bias:

- Men and women aged 18 and over
- Must be able to provide written informed consent
- Must be able to participate in telephone interviews during the study period (most likely during Q1 2022)
- Must have the cognitive and linguistic skills necessary to participate
- Patients with confirmed ATTRv or ATTRwt amyloidosis
- Completed the survey and included information on type of manifestation, age and type of HCP seen for each manifestation

The participants will be given a full written explanation of the study including background, purpose and steps involved in the interview process. Prior to the interview, they will be sent the study informed consent form by e-mail or post. Those who agree to participate will be asked to sign and return the consent form, and a 1- 1.5-hour appointment will be agreed for the interview with a trained BresMed researcher.

Up to 25 patients will be recruited. Due to the small number of patients with ATTR amyloidosis, randomised sampling is not feasible. Therefore, we will make use of purposive sampling with the aim to interview a variety of patients (e.g. in terms of age, age at diagnosis and severity of disease) to allow us to capture a range of experiences of their journey to an ATTR amyloidosis diagnosis. Following initial review of the survey data, this target number is likely feasible.

3.2.2 Interview guide design

The interview guide will be semi-structured to ensure that topics of interest are covered but provide flexibility for the interviewer to probe and explore newly emerging themes, tailoring the questions to the respondent as needed. The topics covered in the interview guide will be based on those identified as particularly important to patients with ATTR amyloidosis based on the survey analysis and discussions between BresMed, the ASG, medical experts and Alnylam Pharmaceuticals. To support patient recall, the BresMed researchers will share individualised patient journey pathways (developed on PowerPoint) mapping the participants journey as reflected from their individual survey results. This will be shared to the participants prior to the interview giving them time to

review it, discuss it with family or caregivers who were supportive during their ATTR amyloidosis diagnosis to reduce recall bias. The journey pathways will be shared with the participants via PowerPoint/PDF and presented during the interview to help structure the interviews.

Prior to the start of the interviews, an internal pilot interview will be conducted with a BresMed researcher not involved in the project to test the structure, clarity and comprehensibility of the patient questions as well as the length of the interview. It is recommended that one external pilot interview with a patient is also conducted and if possible, feedback will be attained from Alnylam's patient panel, a group of patients with ATTR amyloidosis that meet regularly to review such materials and provide improvements based on their experiences of the condition. After each pilot phase, the interview guides may be slightly adapted to further refine the questions and correct any risk of bias. The final interview guides will be reviewed and approved by Alnylam Pharmaceuticals.

3.2.3 Interview process

The in-depth interviews will be semi-structured in nature and designed to take a maximum of one and a half hours. The interview guide will form the basic structure of the interviews. However, researchers may deviate from the guides to explore any interesting responses provided by participants to ensure that the qualitative interviews provide a rich insight into the patients' perspectives. To minimise the burden to the patients, BresMed will aim to be as flexible as possible in scheduling the interviews to not hinder the recruitment process.

All interviews will be recorded upon consent from the participant for transcribing and data analysis purposes. If participants do not consent to be recorded, notes will be taken during the interview by a second BresMed researcher. The transcriptions of the interviews will be available only to BresMed for analysis purposes. Adverse events (AEs) will be handled in compliance with Alnylam Pharmaceutical's internal pharmacovigilance reporting requirements. All personal information will be stored and handled in accordance with the general data protection regulation (GDPR).

3.3 Data collection and data storage

For Part 1, data collected using the online survey platform was downloaded and stored by BresMed on its secure password protected server for data cleaning and analysis. Personal identifiable information collected in the survey (e.g., email address, IP address) was removed by the BresMed team to prepare an anonymized data set to be shared with Alnylam for storage on a secure password protected server.

For Part 2, personal identifiable information will also be removed from the interview transcriptions and assigned a unique respondent code. Only the researchers who are part of the BresMed will have access to anonymized individual responses held in secure folders on the respective servers. The data collected will only be used for the objectives of this study (see section 3.4.1).

Aggregated results will be shared with Alnylam, the ASG and KOLs, and may also be used for publication; however, anonymized individual patient level data will remain with the research teams from Alnylam and BresMed only. Please see section 3.5 for further information on confidentiality and data protection.

3.4 Data analysis and reporting

3.4.1 Analysis

Part 1 – Survey:

Due to the unanticipated high enrolment in the survey (>1000 responses between November 30 and December 8, 2020) and uncharacteristic patient demographics (as noted by experienced clinicians and members of the ASG) among survey participants in both hATTR amyloidosis and wtATTR amyloidosis patient groups, particularly in the last 5 days of data collection (Figure 1), it was determined that an additional data cleaning step will be required prior to data analysis to identify ‘true’ patients. A rapid acceleration in patient recruitment and notable change in the average age of participants (i.e., namely, younger participants than is typical for either hATTR or wtATTR amyloidosis) starting on the 5th day of recruitment, Friday November 4, 2020 was observed.

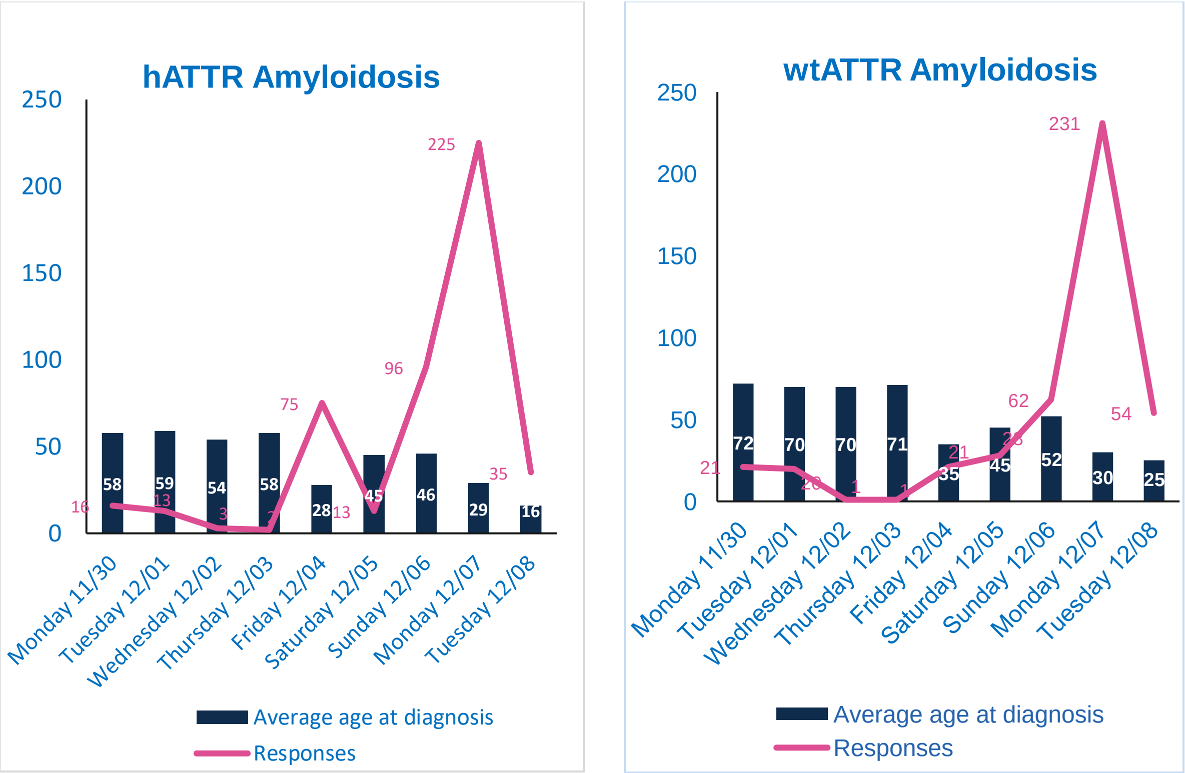

Figure 1. Day-to-day patient enrolment and mean age of participants throughout the survey recruitment period.

To strengthen internal validity, the ASG leadership confirmed each survey subject's patient status as someone living with ATTR amyloidosis before their data was included in the analysis. To accomplish this, BresMed shared the email addresses collected as part of the survey with the ASG leadership; sharing email information from the following two fields of the survey:

1. Emails provided by the participants who consented to be contacted for follow-up in depth interviews
2. Emails provided by the participants who wished to receive the $30 Amazon gift card as compensation for their time completing the survey

The ASG leadership cross referenced these email addresses with their own patient list and flagged those emails corresponding to patients who are known to the ASG patient organization.

Additionally, patients who completed the survey and chose not to disclose their email address, including those not to receive the compensatory Amazon gift card, will be included considered for the analysis (maximum 44 participants). It is considered reasonable by the researchers to consider these participants as likely to be patients living with ATTR amyloidosis as they did not have a financial incentive to participate in the study and were more likely to have participated for altruistic reasons. Patient demographics and disease characteristics for the subgroups ATTRwt and ATTRv amyloidosis will be summarized. Through the expert opinions of the researchers, it will be determined whether this cohort reflects the disease characteristics of subjects included in previous publications of ATTR amyloidosis. The researchers will determine whether or not to include these patients in the analysis cohort.

Additional criteria for data cleaning purposes may be considered, such as excluding:

- Responses that come from the same IP address, provided that the responses suggest that it is the same person answering twice
- Respondents that have not mentioned at least one sign/symptom, procedure or other diagnosis
- Respondents who provided inconsistent answers" (e.g. the 'faulty age' criterion, those that put ages older than their diagnosis age)

In summary, only data from participants who adhere to the inclusion criteria and are a) on the validated patient lists (i.e., confirmed by the ASG) and potentially (pending data review) b) who completed the survey but did not opt-in to receive financial compensation and did not disclose their email addresses for other reasons will be used for survey data analysis (i.e., analysis group).

The anticipated statistical or thematic analysis for each question within the survey will be shared with Alnylam for approval prior to the survey analysis. Due to the second objective being to evaluate the similarities and differences between the two conditions, the results will be separated

per condition and analyzed separately. Following this analysis, the results from the two conditions will be compared to highlight the similarities and differences.

Descriptive statistical analysis will be performed on the close-ended questions. Central tendency (median, mode) and variation (range, interquartile range) will be calculated when appropriate. Additionally, thematic data analysis will be applied to recognize patterns in the qualitative data obtained.

Part 2 – Interviews:

In qualitative research, thematic analysis is frequently used to recognise patterns in the collected data and based on that, test hypotheses and/or form new ideas. These themes can either come from previous research and theories (deductive) or can be newly identified through investigating what themes emerge from the data (inductive), or a combination of both.

For this research, thematic analysis will be used to identify recurring themes, using both inductive and deductive coding methods. A coding framework (i.e., a framework with initially identified themes that will be used for coding the data) will be developed and implemented.

The stages involved in thematic analysis can be broadly divided as follows:

1. Familiarisation with data (becoming thoroughly immersed in the material collected)
2. Developing a thematic framework (identifying key issues from data)
3. Coding the data (labelling key issues that emerge across a set of data)
4. Devising a series of thematic charts (allowing the full pattern across a set of data to be explored and reviewed)
5. Mapping and interpreting data (looking for associations, providing explanations and highlighting key characteristics and ideas)

Data will be managed and analysed using QSR NVivo software, which is specifically designed for the analysis of qualitative data.

3.4.2 Reporting

The final anonymized and aggregated patient results from the survey will be reported within a PowerPoint presentation. This will highlight the key findings of the survey for Alnylam Pharmaceuticals, the ASG, or clinical experts to consider.

A summary report from the qualitative interviews will be generated in a Word document, including a thematic summary of key points raised by patients related to each research objective as well as supporting, de-identified patient quotes.

Any external publication or disclosure to the medical community, health authorities, or patient organizations, if any, will be limited to the anonymized findings of this research.

3.5 Confidentiality and data protection

3.5.1 Confidentiality and data protection

Identifiable data linking the participants to their survey or interview responses will not be shared with Alnylam Pharmaceuticals, the ASG, or clinical experts and will only be accessible to the BresMed team. In summary, participant data will be stored on a secure server and in a password-protected location. BresMed will only share participant email addresses with the ASG for analysis purposes due to the unexpectedly high response rate (please see section 3.3.1). No other data (physical or soft copies) or personally identifiable subject information will be shared outside the BresMed office or folder on the server. Only the research team within BresMed will have access to this folder. Once the project is completed, the data will be stored on a secured file and archived for 3 years. BresMed is trained in upholding General Data Protection Regulation (GDPR) and will not store data on flash drives or computers other than those specific for work. When possible, data will not be stored on individual computers, and access to those computers (that access the server) will be locked in offices overnight. Encrypted and multifactor authentication processes have been put in place that will prevent access to the server to anyone other than BresMed employees.

In detail, to protect the participants' confidentiality, several safeguards will be implemented:

- If a participant decides not to take part in the research, personal identifiable information will only be held where needed for compliance with law. No further data will be retained
- If requested by the participant, any personal identifiable information held by BresMed will be shared with them
- It will be made clear that a participant can opt-out of the survey or have their data excluded from analysis and results by emailing a researcher to request withdrawal of their data from the study
- Only aggregated data will be published. This data cannot be revised should a participant withdraw consent after publication
- During the process of verification of participants' patient status, all personal information (i.e., patient emails) will be shared directly between BresMed and the ASG. Alnylam and the HCP clinical experts will be blinded to all personal data and will not be involved in this additional data cleaning procedure
- All transcripts and publishable quotes will be redacted of any personal identifiable data and not be shared with Alnylam

3.5.2 Adverse event reporting

BresMed must comply with all Adverse Event and Product Complaint reporting obligations under applicable laws in the United States and send copies of all reports (including but not limited to interim study reports, final study reports, etc.) submitted to any health authority or regulatory agency regarding an Alnylam product in parallel to Alnylam via Email: **patientsafety@alnylam.com** or Fax: **1-289-846-5210**.

Additionally, BresMed must report all Adverse Events (AEs) (as defined below) and/or Other Safety Information (as defined below) concerning Alnylam's products to Alnylam Global Patient Safety and Risk Management within 24 hours of first knowledge by BresMed via Email: **patientsafety@alnylam.com** or Fax: **1-289-846-5210**.

BresMed must report all Product Complaints (PCs) (as defined below) via Email: **medinfo@alnylam.com**

BresMed shall ensure that any personal information collected about individuals complies with applicable privacy and data protection laws and regulations.

Definitions:

Adverse Event (AE): Any untoward medical occurrence in a patient administered an Alnylam marketed product and which does not necessarily have a causal relationship with this treatment. An adverse event can therefore be any unfavourable and unintended sign (for example, an abnormal lab finding) symptom, or disease temporally associated with the use of an Alnylam marketed product, whether or not considered related to this Alnylam marketed product.

Other Safety Information: Other Safety Information means circumstances where a report does not include an AE per se, but nevertheless needs to be reported to Alnylam. These circumstances include:

- Use of a product during pregnancy or breastfeeding
- Overdose
- Abuse
- Misuse
- Medication error
- Occupational exposure
- Lack of therapeutic efficacy
- Off-label use
- Suspected transmission via a medicinal product of an infectious agent
- Suspected or confirmed counterfeit/falsified medicinal product

Product Complaint (PC): Any written, electronic, or oral communication that alleges deficiencies related to the identity, quality, durability, reliability, safety, effectiveness, or performance of a medicinal product, medical device, or combination product after it is released for distribution. Product

complaints are typically non-medical in nature; however, it is possible that complaints could be associated with an adverse event.

4. Ethical review

The study protocol, survey and interview questions, and relevant documents including the e-consent and patient recruitment materials will be approved or given favourable opinion in writing by a centralized IRB. This approval must be granted before patients can be enrolled in the survey.

In the event of any changes or amendments to the study protocol in accordance with the requirements, the protocol must be reapproved by the IRB upon receipt of amendments and annually, as local regulations require. Initial IRB approval of the protocol, and all materials approved by the IRB for this study including the e-consent form and recruitment materials, as applicable, will be maintained by Alnylam and will be made available for inspection.

Participation is voluntary. Before starting the survey, participants will be asked to give their consent to share their anonymized results with BresMed Health Solutions which is acting on behalf of a pharmaceutical company. Participants will have the right to withdraw from the research at any time. Once the participant has read and understood the survey objectives, background and their requirements, they will be asked if they agree to consent to the survey. If the participant does not wish to progress and provide consent, or if they are not eligible following the inclusion criteria questions/statement, they will be redirected to a “thank you for considering taking part in our research” page of the survey. In addition, raw data will be stored by BresMed and will only be accessible to BresMed researchers in accordance with applicable data protection laws. Participants will be assigned a unique code which will be used on all their documentation to protect their identity.

4.1 Reimbursement and compensation for research participants

For the survey, participation was voluntary and participants were offered a $30 Amazon gift card as compensation for their time. This wasa short survey online and therefore burden and inconvenience was seen as minimal.

For the qualitative interviews, participation is voluntary, and participants will be offered a $60 Amazon gift card as compensation for their time.

5. References

1. Mohty D, Damy T, Cosnay P, et al. Cardiac amyloidosis: updates in diagnosis and management. *Arch Cardiovasc Dis*. 2013; 106(10):528-40.

2. Hawkins PN, Ando Y, Dispenzeri A, et al. Evolving landscape in the management of transthyretin amyloidosis. *Ann Med*. 2015; 47(8):625-38.

3. Conceição I, González-Duarte A, Obici L, et al. "Red-flag" symptom clusters in transthyretin familial amyloid polyneuropathy. *J Peripher Nerv Syst*. 2016; 21(1):5-9.

4. Shin SC and Robinson-Papp J. Amyloid neuropathies. *Mt Sinai J Med*. 2012; 79(6):733-48.

5. Rapezzi C, Quarta CC, Obici L, et al. Disease profile and differential diagnosis of hereditary transthyretin-related amyloidosis with exclusively cardiac phenotype: an Italian perspective. *Eur Heart J*. 2013; 34(7):520-8.

6. Coelho T, Maurer MS and Suhr OB. THAOS - The Transthyretin Amyloidosis Outcomes Survey: initial report on clinical manifestations in patients with hereditary and wild-type transthyretin amyloidosis. *Curr Med Res Opin*. 2013; 29(1):63-76.

7. Adams D, Gonzalez-Duarte A, O'Riordan WD, et al. Patisiran, an RNAi Therapeutic, for Hereditary Transthyretin Amyloidosis. *N Engl J Med*. 2018; 379(1):11-21.

8. Hanna M. Novel drugs targeting transthyretin amyloidosis. *Curr Heart Fail Rep*. 2014; 11(1):50-7.

9. Castaño A, Drachman BM, Judge D and Maurer MS. Natural history and therapy of TTR-cardiac amyloidosis: emerging disease-modifying therapies from organ transplantation to stabilizer and silencer drugs. *Heart Fail Rev*. 2015; 20(2):163-78.

10. Gertz MA, Kyle RA and Thibodeau SN. Familial amyloidosis: a study of 52 North American-born patients examined during a 30-year period. *Mayo Clin Proc*. 1992; 67(5):428-40.

11. Sattianayagam PT, Hahn AF, Whelan CJ, et al. Cardiac phenotype and clinical outcome of familial amyloid polyneuropathy associated with transthyretin alanine 60 variant. *Eur Heart J*. 2012; 33(9):1120-7.

12. Swiecicki PL, Zhen DB, Mauermann ML, et al. Hereditary ATTR amyloidosis: a single-institution experience with 266 patients. *Amyloid*. 2015; 22(2):123-31.

13. Khella S DB, Damy T et al. Prevalence and severity of neuropathy on THAOS subjects with wild-type and hereditary transthyretin cardiac amyloidosis. *Peripheral Nerve Society (PNS) Baltimore, MD, USA Poster P26*. 2018.

14. Maurer MS, Bokhari S, Damy T, et al. Expert Consensus Recommendations for the Suspicion and Diagnosis of Transthyretin Cardiac Amyloidosis. *Circ Heart Fail*. 2019; 12(9):e006075.

15. Maurer MS, Hanna M, Grogan M, et al. Genotype and Phenotype of Transthyretin Cardiac Amyloidosis: THAOS (Transthyretin Amyloid Outcome Survey). *J Am Coll Cardiol*. 2016; 68(2):161-72.

16. Witteles RM, Bokhari S, Damy T, et al. Screening for Transthyretin Amyloid Cardiomyopathy in Everyday Practice. *JACC Heart Fail*. 2019; 7(8):709-16.

17. Connors LH, Sam F, Skinner M, et al. Heart Failure Resulting From Age-Related Cardiac Amyloid Disease Associated With Wild-Type Transthyretin: A Prospective, Observational Cohort Study. *Circulation*. 2016; 133(3):282-90.

18. Gillmore JD, Damy T, Fontana M, et al. A new staging system for cardiac transthyretin amyloidosis. *Eur Heart J*. 2018; 39(30):2799-806.

19. Givens RC, Russo C, Green P and Maurer MS. Comparison of cardiac amyloidosis due to wild-type and V122I transthyretin in older adults referred to an academic medical center. *Aging health*. 2013; 9(2):229-35.

20. Grogan M, Scott CG, Kyle RA, et al. Natural History of Wild-Type Transthyretin Cardiac Amyloidosis and Risk Stratification Using a Novel Staging System. *J Am Coll Cardiol*. 2016; 68(10):1014-20.

21. Pinney JH, Whelan CJ, Petrie A, et al. Senile systemic amyloidosis: clinical features at presentation and outcome. *J Am Heart Assoc*. 2013; 2(2):e000098.

22. Ruberg FL, Maurer MS, Judge DP, et al. Prospective evaluation of the morbidity and mortality of wild-type and V122I mutant transthyretin amyloid cardiomyopathy: the Transthyretin Amyloidosis Cardiac Study (TRACS). *Am Heart J*. 2012; 164(2):222-8.e1.

23. Siepen FAD, Bauer R, Voss A, et al. Predictors of survival stratification in patients with wild-type cardiac amyloidosis. *Clin Res Cardiol*. 2018; 107(2):158-69.

24. Plante-Bordeneuve V. Update in the diagnosis and management of transthyretin familial amyloid polyneuropathy. *J Neurol*. 2014; 261(6):1227-33.

25. Ando Y, Coelho T, Berk JL, et al. Guideline of transthyretin-related hereditary amyloidosis for clinicians. *Orphanet J Rare Dis*. 2013; 8:31.

26. Connors LH, Doros G, Sam F, et al. Clinical features and survival in senile systemic amyloidosis: comparison to familial transthyretin cardiomyopathy. *Amyloid*. 2011; 18 Suppl 1(Suppl 1):157-9.

27. Falk RH, Kruger, J. & Quarta, C. C. Senile systemic amyloidosis is a markedly underdiagnosed cardiomyopathy: experience of a cardiac amyloidosis program [abstract]. *J Am Coll Cardiol 61 (Suppl 10), E1241 (2013)*. 2013.

28. Kittleson MM, Maurer MS, Ambardekar AV, et al. Cardiac Amyloidosis: Evolving Diagnosis and Management: A Scientific Statement From the American Heart Association. *Circulation*. 2020; 142(1):e7-e22.

29. Isabelle Lousada MSM, Melissa Warner, Spencer Guthrie, Kristen Hsu, Martha Grogan. Amyloidosis Research Consortium Cardiac Amyloidosis Survey: Results from Patients with AL and ATTR Amyloidosis and Their Caregivers. Presented at the 23rd annual Heart Failure Society of America; September 13-16, 2019; Philadelphia, PA 2019.

30. Ruberg FL, Grogan M, Hanna M, et al. Transthyretin Amyloid Cardiomyopathy: JACC State-of-the-Art Review. *J Am Coll Cardiol*. 2019; 73(22):2872-91.

31. Halatchev IG, Zheng J and Ou J. Wild-type transthyretin cardiac amyloidosis (ATTRwt-CA), previously known as senile cardiac amyloidosis: clinical presentation, diagnosis, management and emerging therapies. *J Thorac Dis*. 2018; 10(3):2034-45.

32. González-López E, Gagliardi C, Dominguez F, et al. Clinical characteristics of wild-type transthyretin cardiac amyloidosis: disproving myths. *Eur Heart J*. 2017; 38(24):1895-904.

33. Trivedi JR, Cannon SC and Griggs RC. Nondystrophic myotonia: challenges and future directions. *Exp Neurol*. 2014; 253:28-30.

34. Gertz MA, Benson MD, Dyck PJ, et al. Diagnosis, Prognosis, and Therapy of Transthyretin Amyloidosis. *J Am Coll Cardiol*. 2015; 66(21):2451-66.

35. Benson MD, Waddington-Cruz M, Berk JL, et al. Inotersen Treatment for Patients with Hereditary Transthyretin Amyloidosis. *N Engl J Med*. 2018; 379(1):22-31.

36. Grogan M, Hawkins PN, Kristen AV, et al. Identifying Mixed Phenotype: Evaluating the Presence of Polyneuropathy in Patients with Hereditary Transthyretin-Mediated Amyloidosis with Cardiomyopathy. 2019 Heart Failure Society of America (HFSA) Annual Scientific Meeting. 2019/08/01/ 2019.
