# Supplementary material for: The journey to diagnosis of wild-type transthyretin-mediated (ATTRwt) amyloidosis: a path with multisystem involvement
Source: Orphanet J Rare Dis. 2024 Nov 8;19:419. doi: 10.1186/s13023-024-03407-3 (PMC11549766; doi:10.1186/s13023-024-03407-3)
Supplement: Supplementary file 2 — Additional file 2: Interview guide. [file 13023_2024_3407_MOESM2_ESM.pdf]

Contents

Abbreviations **1**

1. Introduction **3**

2. Interview guide **5**

2.1. Introductory questions 5

2.2. Understanding the patient experiences from their starting point [*X*] *years/over 10 years* prior to their ATTR amyloidosis diagnosis 7

2.3. 10 - 4 years prior to your ATTR amyloidosis diagnosis 7

2.4. 3 - 0 years prior to your ATTR amyloidosis diagnosis 8

2.5. Current management and reflection since your ATTR amyloidosis diagnosis 9

2.6. Support from family, caregivers or organisations 10

3. Thank you – End of interview **11**

Abbreviations	Definition
ASG	Amyloidosis Support Groups
ATTR amyloidosis	Transthyretin-mediated amyloidosis
HCP	Healthcare provider (includes anyone providing medical care e.g. a medical specialist, psychologist, or an infusion nurse)

1. Introduction

- *Interviewer introductions*

Lumanity (previously BresMed Health Solutions) is working on behalf of a pharmaceutical company to conduct interviews to explore the journey to diagnosis of patients with transthyretin-mediated (ATTR) amyloidosis. We would like to understand your journey from the moment you first experienced symptoms associated with ATTR amyloidosis (either known to be due to ATTR amyloidosis at the time or retrospectively associated with your ATTR amyloidosis) up until you received your formal diagnosis, and how this journey impacted you at the time, such as in your work, social life as well as physically and emotionally. We would also like to understand which health care practitioners were involved during your journey to diagnosis, and how your interactions with them help address unmet needs and/or assisted in reaching your diagnosis of ATTR amyloidosis.

Please note that this discussion is designed for research only. This means that nothing is being promoted or sold to you during this discussion. Any information discussed during the research should not be used to influence decisions outside these conversations.

There are no expected medical benefits to you for taking part in this study. The anonymized results of the interviews will help us understand common or unique milestones in the journey to ATTR amyloidosis diagnosis. We hope our results will help identify key decision triggers and/or unmet needs that can improve the time to ATTR amyloidosis diagnosis for future patients. We hope to communicate our findings to health insurance providers, medical professionals, and the broader community to support faster diagnosis times.

You should have been approached over the telephone or via email by one of our Lumanity researchers regarding participating in this research. Hopefully, you have been given a full written explanation of the study, and a consent form to read and sign. Is this correct?

Q: Do you have any questions about any of these materials?

While we have developed the questions with great care, some questions may feel personal or emotional. If you want to take a break at any point during the interview or do not feel comfortable answering a particular question, please do not hesitate to let me know.

Q: Do you have any questions or concerns about this research?

So that we don't miss anything important, it would be helpful for us if we can record the interview. To protect your anonymity, the transcripts and recordings will be stored by Lumanity and will only be accessible to Lumanity researchers in accordance with applicable data protection laws.

Q: Do we have your consent to record this interview?

[If yes] *begin recording and ask question again to tape the respondent's confirmation*

[If no] *confirm if we can take notes (if not, thank the interviewee and end the interview)*

Before we start, I am required to read a safeguarding statement and a medicine safety statement to you.

Personal information

Lumanity will not store data on flash drives or computers other than those specific for work. When possible, data will not be stored on individual computers, and access to those computers (that access the server) will be locked in offices overnight. Encrypted and multifactor authentication processes have been put in place that will prevent access to the server to anyone other than Lumanity employees. Please note your personal information will not be sold nor shared with any third parties for advertising purposes.

Safeguarding

If it is evident someone is suffering (emotionally or physically) or is likely to suffer significant harm, a referral must be made to the appropriate authority without delay in order that the individual can be protected. The interviewer must ensure that any closure or of confidential nature, which may be potentially harmful to the individual, must be dealt with in a sensitive and responsible manner.

Medicine Safety

While what we discuss during the interview is confidential, Lumanity is obligated to disclose to the Sponsor, any adverse events, other safety information and/or product complaints that you may mention about any of the Sponsor's products. Lumanity will ask you for your consent to share your identifiable information with the sponsoring company if you mention any adverse event, other safety information and/or product complaint about any of the sponsor's product. If you do not consent to sharing your identifiable information, you can still participate in this interview and the

adverse event, other safety information and/or product complaint will be provided anonymously to the sponsoring company and the sponsoring company will not be able to reach out to you for additional information. If you consent for us to share your identifiable information under these circumstances, a representative from the Pharmacovigilance department of the sponsoring company may reach out to you to collect additional information.

Everything else you mention in this interview will continue to remain confidential.

Q: Are you agreeable to proceed with the interview on this basis?

If yes, start interview

- *During the interview, wording used by the respondent to describe their experiences, milestones etc. will be used throughout the interview by the interviewer as much as possible*
- *The interview guide serves as a guide:*
    - *The individual respondent's patient journey PowerPoint slide will be used to help guide the interview, ensuring the discussion is more tailored to the respondent*
    - *If multiple questions are covered by the respondent in one go, the interviewer may skip questions. In addition, the interviewer may ask probing questions to better understand the respondent's answers*

We shared with you, your survey results in your personalised patient journey with a request to check it for accuracy. We aim to use this [updated] version you shared throughout the interview. Before sharing the map on the screen, I just have a few questions before the start of your ATTR amyloidosis diagnosis journey.

2. Interview guide

During this interview, we will start with some general questions about yourself, followed by a more detailed discussion of your journey to your ATTR amyloidosis diagnosis. We will end by giving you the opportunity to talk about any topics that we did not cover in the interview.

2.1. Introductory questions

1. Could you tell me a little bit about yourself?

a. Do you mind if I ask you how old you are?

b. What is your family situation? (Prompt: married/partner/single; children/grandchildren)

c. Are you currently employed, retired, in voluntary work, or education?

Before the start of your journey to ATTR amyloidosis diagnosis began:

2. What specialists did you see on a regular basis? [Prompt: How regularly did you see your primary care physician or have regular general check-ups? What other HCPs did you see? Did you see any HCPs for any mental health issues?]

3. Did you experience any other diagnosed health conditions before your ATTR amyloidosis symptoms started? [If yes, prompt: what other diagnosed health conditions and/or procedures did you have?]

The next part of the interview aims at understanding your journey to a diagnosis with ATTR amyloidosis. We will do this by going through the slide we shared with you that summarises your personal experiences and diagnostic journey in a chronological order, looking at the period: when you first started experiencing symptoms, the period [*X years / over 10 years*] prior to diagnosis (if applicable), followed by the period 10 - 4 years before diagnosis, and finally 3 – 0 years leading up to your diagnosis.

Thank you for providing your [updated] journey with us, you mentioned in the survey that you:

- Were diagnosed with ATTR amyloidosis at [*age*].
- Started experiencing symptoms at [*age*], [*X*] years before your ATTR amyloidosis diagnosis.
- [If applicable]: Received a genetic test at age [*X*] before your ATTR amyloidosis at age [*X*]. [Prompt: What led you to have a genetic test? Were there any challenges to arranging your genetic test?]

4. Is this correct?

Mapping the patient's diagnostic journey *[present journey on the zoom screen]*

5. Please describe your earliest memory of experiencing symptoms, or first memorable health event, that started your journey to a ATTR amyloidosis diagnosis.

6. Did you seek medical attention at that time?

a. If yes, what HCP(s) did you visit?

    i. What was the deciding factor for reaching out to an HCP about your symptoms? [prompt: e.g., that moment where you went 'ah, okay, I need to see my physician now'?]

b. If no, could you explain why not? [Prompt: Did you end up seeing an HCP for these symptoms? What made you visit an HCP later? How did you treat / manage these symptoms?]

[*Depending on individual patient journey maps, adjust the time points in the interview with the time points provided by the participant and then move onto the relevant chapter thereafter]*

2.2. Understanding the patient experiences from their starting point [*X] years/over 10 years* prior to their ATTR amyloidosis diagnosis

7. [*During X years / 'Over 10 years'*] prior to your ATTR amyloidosis diagnosis, you stated you experienced [*list symptoms, procedures, and other diagnoses*]. Is that correct?

8. When thinking back to this time, what was your daily life like?

a. What impact did *[x]* symptom/procedure/other diagnosis have on you being able to carry out certain activities, work, financial, family situation, vacations, hobbies etc.?

b. *Only* if they experienced a big health event: What changes occurred in your life due to this event? E.g. what could you do or not do in your daily life?

c. Which symptoms, procedures or other diagnoses impacted you most during this time of your life? [Prompt: activities carried out, work, family situation, vacations, hobbies etc.]

d. How did this/these events impact you emotionally? [Prompt: What emotions did you feel at the time? What emotions come up now thinking back to that time in your life?]

9. What were the key turning points during this time in your life? [Prompt: Please share any key experiences that upon reflection were trigger points for change within your health care at that time? For example, any 'ah ha moments', did you change HCP, or experience a key event/other diagnosis or procedure or change any personal behaviour/activities over time?]

a. What would you have liked to have happen differently if anything?

10. Thank you for providing your experiences during this period. Are there any additional comments you'd like to mention that was important to you at that time that you feel is an important part of your journey or you emotional and physical wellbeing?

2.3. 10 - 4 years prior to your ATTR amyloidosis diagnosis

11. You mentioned you experienced [*list symptoms*], [*X years / over 10 years*] prior to diagnosis. Did these symptoms continue throughout your journey to an ATTR amyloidosis diagnosis?

a. If not, how were they resolved? [Prompt: did you keep going back to see the same HCPs? If so, which ones?]

b. If they persisted, did these symptoms change (if at all)? [prompt: did they got worse, or better over time – if better, how come (treatment etc.)].

12. You also stated you experienced new symptoms [list symptoms] during 10 – 4 years prior to ATTR amyloidosis diagnosis.

a. Did you experience any other symptoms/procedures/other diagnoses that are missing from this list?

b. Did any of these symptoms change over time? [prompt: did they get worse, or better over time – if better, how come (treatment etc.)].

Understanding the patient experiences 10 - 4 years prior to their ATTR amyloidosis diagnosis

13. How did your daily life change during this time of your journey to a ATTR amyloidosis diagnosis?

a. What impact did *[x]* symptom/procedure/other diagnosis have on you being able to carry out certain activities, work, financial, family situation, vacations, hobbies etc.?

b. *Only* if they experienced a big health event: What changes occurred in your life due to this event? E.g. what could you do or not do in your daily life?

c. Which symptoms, procedures or other diagnoses impacted you most during this time of your life? [Prompt: activities carried out, work, family situation, vacations, hobbies etc.]

d. How did this/these events impact you emotionally? [Prompt: What emotions did you feel at the time? What emotions come up now thinking back to that time in your life?]

14. What were the key turning points during this time in your life? [Prompt: Please share any key experiences that upon reflection were trigger points for change within your health care at that time? For example, any ‘ah ha moments’, did you change HCP, or experience a key event/other diagnosis or procedure or change any personal behavior/activities over time?]

a. What would you have liked to have happen differently if anything?

2.4. 3 - 0 years prior to your ATTR amyloidosis diagnosis

15. You mentioned you experienced [*List symptoms*] over 10 - 4 years prior to diagnosis. Moving on to the period 3 – 0 years prior to diagnosis, did these symptoms carry on at this time?

a. If not, how were they resolved?

b. If they persisted, did these symptoms change (if at all)? [Prompt: did they got worse, or better over time – if better, how come (treatment etc.)].

16. You also stated you experienced new symptoms, procedures and other diagnoses *[list]* in 3 – 0 years prior to ATTR amyloidosis diagnosis.

a. Did you experience any other symptoms/procedures/other diagnoses that are missing from this list?

b. Did any of these symptoms change over time? [Prompt: did they got worse, or better over time – if better, how come (treatment etc.)].

Understanding the patient experiences 3 - 0 years prior to their ATTR amyloidosis diagnosis

Given the changes in your symptoms, to what extend did the impact on your daily life change at this time? [Prompt: was it the same as before or were there any key milestones that you remember]

17. How did your daily life change during this time of your journey to a ATTR amyloidosis diagnosis?

a. What impact did *[x]* symptom/procedure/other diagnosis have on you being able to carry out certain activities, work, financial, family situation, vacations, hobbies etc.?

b. *Only* if they experienced a big health event: What changes occurred in your life due to this event? E.g. what could you do or not do in your daily life?

c. Which symptoms, procedures or other diagnoses impacted you most during this time of your life? [Prompt: activities carried out, work, family situation, vacations, hobbies etc.]

d. How did this/these events impact you emotionally? [Prompt: What emotions did you feel at the time? What emotions come up now thinking back to that time in your life?]

18. What were the key turning points during this time in your life? [Prompt: Please share any key experiences that upon reflection were trigger points for change within your health care at that time? For example, any 'ah ha moments', did you change HCP, or experience a key event/other diagnosis or procedure or change any personal behavior/activities over time?]

a. What would you have liked to have happen differently if anything? [Prompt: If you could have been diagnosed/treated sooner, what impact would this have had in your daily life?]

2.5. Current management and reflection since your ATTR amyloidosis diagnosis

Following your ATTR amyloidosis diagnosis, we'd like to ask questions about how your diagnostic journey could potentially have been improved and how ATTR amyloidosis affects you now.

19. Following your ATTR amyloidosis diagnosis, did you go back to your previous physicians and let them know about your ATTR amyloidosis diagnosis?

20. How much coordination or organization was involved for your care throughout your journey to diagnosis? [Prompt: what was involved in terms of who you spoke to, when and how relating to your treatment/care or monitoring?]

a. Did this change over time? [Prompt: If so, how? E.g., did you have the same cardiologist throughout your journey or multiple different cardiologists?]

b. Are there any aspects of your care or communication with any of your HCPs that you would have liked to have seen differently? [Prompt: What would you have liked to have seen be done differently?]

i. Were there any ‘red flags’ that were missed?

21. How (if at all) were you able to keep track of your symptoms, doctor visits etc.? [Prompt: Was it all done for you by your main HCP, or did a loved one/informal caregiver help keep track?]

a. Did this change throughout your journey to your ATTR amyloidosis diagnosis?

b. What could have helped you keep track from the earliest symptoms, procedures or other diagnoses e.g., a journal/digital app?

22. In retrospect and thinking about who you are now, what would have supported you most in helping you get diagnosed with ATTR sooner, or more efficiently? [Prompt: Any unmet needs or turning points that could have helped diagnose you sooner?]

a. What could HCPs have done? [Prompt: What would your recommendations to HCPs be for current or future patients in the process of getting diagnosed with ATTR amyloidosis?]

b. What about your medical insurers? [Prompt: What additional support would have helped you when navigating the insurance process?]

c. What about the medical community or any patient association you are/were a member of at that time?

23. Thinking about your current situation, how does ATTR amyloidosis impact you?

a. What symptoms do you still experience?

b. [If they have newer symptoms] What are your first thoughts and consequent actions following the development of new symptoms? e.g., do you go straight to your main HCP or treat at home?

i. When would you consider alerting your HCP?

ii. And how would you define your severity levels?

c. [if appropriate] Do you still experience symptoms related to cardiomyopathy (any issues with the heart muscle e.g., irregular heartbeats) and/or polyneuropathy (e.g., numbness, tingling or unusual sensations/discomfort in hands and/or feet)

d. Which symptoms or aspects of your ATTR amyloidosis impact you the most? [Prompt: Please give examples.]

24. How do you currently manage your ATTR amyloidosis?

2.6. Support from family, caregivers or organizations

25. What kind of support (if any), did you receive from family and friends during your journey to ATTR amyloidosis? [Prompt: What level of support did you need? E.g., bathing, shopping, lifting heavy objects, walking etc.]

26. Did you have an informal (unpaid) caregiver?

  a. What is your relationship to them?

27. Did the level of support differ between [*X years / over 10 years*], 10 - 4 or 3 - 0 years prior to your ATTR amyloidosis diagnosis/did the level of support change over time?

28. Was there any support you needed or would have liked, but did not get? [If yes, prompt: What support would you have needed/liked]

29. From your perspective, how did your ATTR amyloidosis diagnosis impact your loved one[s] or informal caregiver[s]?

a. To what extend does it impact your relationship with them?

30. Did [*insert family member or caregiver name*] notice any early symptoms, changes in attitudes or behavior prior to you reaching out to them or an HCP?

31. We have discussed the support you received from your loved one[s]. What about any professional support, for example, from patient support groups (Amyloidosis Support Groups (ASG) or Amyloidosis Foundation (AF)), or HCPs?

32. [Only ask if relevant]: Specifically thinking about patient support groups, how involved were you during your journey to your ATTR amyloidosis diagnosis?

a. How did you find out about them?

b. Were there any specific services or programs that you found helpful?

c. What services or programs do you think patient support groups should do more of?

d. Did you ever do any internet searches e.g., Google? [If so, prompt: how helpful was it?]

3. Thank you – End of interview

33. Are there any topics that we did not cover during this interview that play an important role on how you came to be diagnosed with ATTR amyloidosis?

34. Are there any areas of impact due to your ATTR amyloidosis diagnosis or journey to your diagnosis that we've not covered that you feel is important?

35. If we have any follow-up questions, could we contact you again?

36. Do you have any more questions for me?

The interviewer will close the interview and thank the respondent for their time.
