# Supplementary material for: The journey to diagnosis of wild-type transthyretin-mediated (ATTRwt) amyloidosis: a path with multisystem involvement
Source: Orphanet J Rare Dis. 2024 Nov 8;19:419. doi: 10.1186/s13023-024-03407-3 (PMC11549766; doi:10.1186/s13023-024-03407-3)
Supplement: Supplementary file 3 — Additional file 3: Patient journal map template. [file 13023_2024_3407_MOESM3_ESM.pdf]

TEMPLATE

Your journey to your wtATTR diagnosis

Patient name

Signs / Symptoms

Procedures

Other diagnoses

HCP visited

Age at manifestation

wATTR

>10 years before diagnosis | 10-4 years before diagnosis | 3-0 years before diagnosis

>10 years before diagnosis	10-4 years before diagnosis	3-0 years before diagnosis
**Signs / symptoms**	**Signs / symptoms**	**Signs / symptoms**
**Procedures**	**Procedures**	**Procedures**
**Other diagnoses**	**Other diagnoses**	**Other diagnoses**

TIP: *It may help to share this map with your loved ones who were with you during your diagnosis journey as they may be able to help you remember some experiences*

BresMed™
