# Supplementary material for: The journey to diagnosis of wild-type transthyretin-mediated (ATTRwt) amyloidosis: a path with multisystem involvement
Source: Orphanet J Rare Dis. 2024 Nov 8;19:419. doi: 10.1186/s13023-024-03407-3 (PMC11549766; doi:10.1186/s13023-024-03407-3)
Supplement: Supplementary file 4 — Additional file 4: List of procedures and symptoms given to participants. [file 13023_2024_3407_MOESM4_ESM.pdf]

List of procedures and symptoms, along with associated survey questions used by participants to report the age at which they have experienced (if at all) symptom suspected to be related to amyloidosis or procedure relating to this potential symptom.

A. *How old were you when you first underwent each procedure? If you have had the procedure more than once, please share how old you were when you first had the procedure. Remember, if you are unsure of your exact age, please provide your best guess*.

Cardioversion
Cardiac pacemaker implantation
Implantable cardioverter defibrillator implantation
Cardiac ablation procedure
Aortic valve replacement surgery
Carpal tunnel release surgery in one wrist
Carpal tunnel release surgery in both wrists (bilateral)
Cubital tunnel release surgery in one elbow
Cubital tunnel release surgery in both elbows (bilateral)
Trigger finger release surgery
Hip replacement surgery
Knee replacement surgery
Shoulder repair surgery
Biceps tendon rupture repair surgery
Hearing aid placement
Spinal fusion surgery
Other spinal surgery
Nerve release surgery

B. *How old were you when you first experienced each sign or symptom? If you've experienced the symptoms more than once, please share how old you were when you first experienced the symptoms. If you are unsure of your exact age, please provide your best guess*

Heart palpitations / heart fluttering
Shortness of breath
Fatigue
Swelling in your lower legs/feet (edema)
Fainting
Dizziness / lightheadedness when changing positions
Numbness in your hands
Numbness in your feet
Tingling like pins and needles in your hands
Tingling like pins and needles in your feet
Unusual sensation or discomfort in your hands
Unusual sensation or discomfort in your feet
Electric shocks
Superficial pain (pain on the surface of your skin)
Deep pain (pain felt deep below your skin)
Loss of pain sensation
Loss of ability to sense temperature (unable to sense when objects are hot or cold)

Unable to regulate body temperature (heat intolerance or cold intolerance)
Muscle weakness
Deadened toe
Diarrhea
Constipation
Alternating constipation and diarrhea
Early fullness (satiety)
Nausea
Vomiting
Abnormal sweating
If male: erectile dysfunction
If female: sexual dysfunction
Voice changes
Loss of bowel control (bowel incontinence)
Loss of bladder control (urinary incontinence)
Difficulty walking
Difficulty swallowing / Choking
